# Benchmarking small-variant genotyping in polyploids

**DOI:** 10.1101/gr.275579.121

**Published:** 2022-02

**Authors:** Daniel P. Cooke, David C. Wedge, Gerton Lunter

**Affiliations:** 1 MRC Weatherall Institute of Molecular Medicine, University of Oxford, Oxford OX3 9DS, United Kingdom;; 2 Manchester Cancer Research Centre, University of Manchester, Manchester M20 4GJ, United Kingdom;; 3 Department of Epidemiology, University Medical Center Groningen, 9713 GZ Groningen, The Netherlands

## Abstract

Genotyping from sequencing is the basis of emerging strategies in the molecular breeding of polyploid plants. However, compared with the situation for diploids, in which genotyping accuracies are confidently determined with comprehensive benchmarks, polyploids have been neglected; there are no benchmarks measuring genotyping error rates for small variants using real sequencing reads. We previously introduced a variant calling method, Octopus, that accurately calls germline variants in diploids and somatic mutations in tumors. Here, we evaluate Octopus and other popular tools on whole-genome tetraploid and hexaploid data sets created using in silico mixtures of diploid Genome in a Bottle (GIAB) samples. We find that genotyping errors are abundant for typical sequencing depths but that Octopus makes 25% fewer errors than other methods on average. We supplement our benchmarks with concordance analysis in real autotriploid banana data sets.

Polyploidy is common in many plant species, including important agricultural crops such as wheat, potato, oat, coffee, rapeseed, cotton, banana, and sugar cane (Song et al. 2012). In mammals, polyploidization regularly occurs during tumorigenesis but has also been shown to be a normal part of development in some mouse and human tissues (Velicky et al. 2018). Molecular markers have been widely used for decades in artificial polyploid crop breeding to assist in selection of more desirable traits such as better resilience to climate change and disease. More recently, genotyping by sequencing has been applied for marker-assisted and genomic selection (He et al. 2014; Kim et al. 2016; Hickey et al. 2019), and the assembly of high-quality plant reference genomes (Jackson et al. 2011; Potato Genome Sequencing Consortium 2011; D'Hont et al. 2012; International Wheat Genome Sequencing Consortium [IWGSC] et al. 2018; Zhuang et al. 2019), together with developments in resequencing, promises new strategies for quantitative trait analysis with a wider variety of genetic variants and better linkage information than is currently possible (Jackson et al. 2011; Uitdewilligen et al. 2013; Bourke et al. 2018; Kyriakidou et al. 2018).

Despite these advances, methods for genotyping polyploids from sequencing data have received little scrutiny in comparison to those for diploids (Bourke et al. 2018; Zook et al. 2019; Li et al. 2018; Krusche et al. 2019). Variant calling and genotyping in polyploids are more difficult than in diploids primarily because the number of possible genotypes at a given loci is combinatorial in the ploidy and number of distinct alleles, and a sequencing read cannot distinguish identical allele copies in the absence of physical linkage with other heterozygous alleles. It therefore becomes harder to determine the allele-specific copy number for a fixed read depth as the ploidy increases. The lower per-allele coverage also makes differentiating true variation from sequencing error less certain. Haplotype-based methods increase power to genotype individual alleles by jointly evaluating combinations of several proximal alleles (haplotypes). They are now standard for diploid calling (Garrison and Marth 2012; Rimmer et al. 2014; Poplin et al. 2017, 2018; Kim et al. 2018; Cooke et al. 2021) and are becoming more common for somatic mutation calling in tumors (Cooke et al. 2021). Unfortunately, only a minority are capable of polyploid calling (Garrison and Marth 2012; Poplin et al. 2017; Cooke et al. 2021), and none have been rigorously tested for this purpose. Specialized methods for polyploid genotyping have been developed (Blischak et al. 2018; Gerard et al. 2018; Clark et al. 2019) but are only suitable for biallelic SNPs. Furthermore, existing benchmarks of polyploid-calling methods fall short of the standard demanded for diploid calling (Uitdewilligen et al. 2013; Clevenger et al. 2015; Krusche et al. 2019; Yao et al. 2020). In particular, we are not aware of any that consider insertions and deletions (indels), genotyping errors in real sequencing data, or representation differences between callers (Krusche et al. 2019). Polyploid genotyping error rates from sequencing are therefore highly uncertain, undermining developments that depends on them.

We sought to address some of these issues by conducting an in-depth assessment of polyploid small variant calling using an independent and comprehensive ground truth, real sequencing data, and haplotype-aware comparisons.

## Results

### Synthetic polyploid genomes

We created synthetic tetraploid and hexaploid samples with high-quality truth sets by merging GIAB v4.2.1 (Zook et al. 2019) GRCh38 variants for the human diploid samples HG002, HG003, and HG004. We chose HG003 and HG004 for the tetraploid sample: the two unrelated parents of HG002. Evaluation regions were defined by intersecting (Quinlan and Hall 2010) the GIAB high-confidence regions for each sample, resulting in 2.50-Gb (86% non-N primary reference) confident tetraploid bases containing 5*,*010*,*307 variants and 2.48-Gb (85% non-N primary reference) confident hexaploid bases containing 4*,*951*,*498 variants. We constructed polyploid Illumina NovaSeq whole-genome test data by mixing reads generated independently for each sample with consistent PCR-free library preparation and depths (Methods). Each individual sequencing run targeted 35× coverage, resulting in 70× coverage tetraploid samples and 105× coverage hexaploid samples. We confirmed total read counts were similar for each contributing sample, ensuring realistic heterozygous allele frequencies. We then randomly down-sampled the full data sets, starting from 10× in 10× intervals to the full coverage, resulting in 6 + 10 = 16 polyploid data sets. All reads were mapped to GRCh38 with BWA-MEM (Methods) (Li 2013).

### Polyploid genotyping accuracy from short-read whole-genome sequencing

We evaluated three popular germline variant callers that support polyploid genotypes—Octopus (Cooke et al. 2021), GATK4 (Poplin et al. 2017), and FreeBayes (Garrison and Marth 2012)—on all synthetic polyploid Illumina data sets, as well as in the diploid HG002 sample to get performance baselines. Other notable germline callers, such as DeepVariant (Poplin et al. 2018), Strelka2 (Kim et al. 2018), and Platypus (Rimmer et al. 2014) were not included because they do not support polyploid calling. We also ignored methods that call polyploid SNVs but not indels, such as polyRAD (Clark et al. 2019). Other than specifying the ploidy and requesting genotype qualities from FreeBayes, we used default setting for all callers (Methods). Octopus calls were hard-filtered with default thresholds; GATK4 and FreeBayes calls were hard-filtered using recommended thresholds (Methods). Variants were compared using RTG Tools vcfeval (Cleary et al. 2015) based on both genotype and allele matches (Methods).

**Figure 1.**
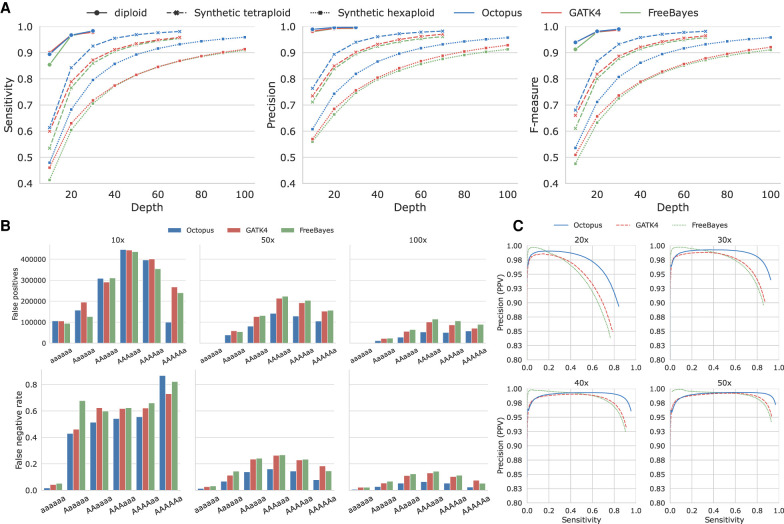
Genotyping accuracy in synthetic polyploids. (*A*) Sensitivity and precision by depth for each caller on real diploid, as well as synthetic tetraploid and hexaploid Illumina data sets. (*B*) Counts of false-positive biallelic calls stratified by depth and genotype (*top*). False-negative rates at biallelic sites stratified by depth and genotype (*bottom*). (*C*) Precision-recall curves for a various tetraploid sequencing depths. Score metrics used to generate the curves were RFGQ (Octopus), GQ (GATK4), and GQ (FreeBayes).

Genotyping accuracy was considerably worse for polyploids compared with diploids (Fig. 1A; Supplemental Table S1). For 30× sequencing depth, on average one of 200 diploid genotype calls were incorrect, in contrast with one of 11 for tetraploid and one of six for hexaploid. Sensitivity was similarly affected; there were 8× and 16× more false negatives on average for tetraploid and hexaploid, respectively, compared with diploid, for 30× sequencing. There were also more substantial differences in accuracy between callers for polyploids compared with diploids. Sensitivity was greater for SNVs than indels, and there was greater disparity between callers for indels (Supplemental Fig. S1; Supplemental Table S2). Overall, Octopus made 26% fewer errors than GATK4 and 30% fewer errors than FreeBayes. However, performance differences varied across depths; the largest F-measure difference between callers occurred at moderate sequencing depth: 30× for tetraploid, 50× for hexaploid. The F-measure showed a typical logarithmic relationship with sequencing depth for both tetraploid and hexaploid samples but also showed a suboptimal response considering ploidy; the F-measure lost from doubling the ploidy was not recovered by doubling the depth, and the differential increased with depth. Stratifying evaluation by GIAB/GA4GH “difficult” regions showed similar results to those found genome-wide (Supplemental Fig. S2; Supplemental Table S1).

Most false-positive genotype calls resulted from calling the incorrect number of variant alleles (allele-specific copy number error): 89% of false-positive biallelic genotype calls (97% of all false positives) were owing to genotyping errors. The most common false positive for all depths was the balanced heterozygotes: AAaa and AAAaaa (Fig. 1B; Supplemental Fig. S3; Supplemental Table S3), 94% of which were owing to incorrect variant allele copy number. A larger fraction of these was made when the true genotype had a −1 variant allele copy rather than a +1 copy (65% vs. 34%) (Supplemental Figs. S4, S5; Supplemental Table S4). The most common biallelic false negatives in tetraploids were simplex heterozygotes (those with a single variant allele copy), whereas for hexaploids, it was duplex heterozygotes (Supplemental Figs. S3, S4). However, normalizing by the true prevalence shows that the most frequent false negative for depths of 30× or more is the balanced heterozygote—the point of maximal variance for binomial distributed allele observations—for both tetraploid and hexaploid; for depths of 20× or more, the most frequent false negative was simplex (Fig. 1B). Furthermore, there was a slight tendency to miscall balanced heterozygotes by a −1 variant allele copy rather than a +1 copy for all callers (Supplemental Figs. S4, S5).

Genotype quality scores were generally well calibrated for all callers (Fig. 1C; Supplemental Fig. S6). However, filtering did not always improve the F-measure; the average F-measure percentage change for filtered versus unfiltered calls on all tests was −0.1%, −0.2%, and +3.5% for Octopus, GATK4, and FreeBayes, respectively. Performance differentials between callers were similar for unfiltered calls (Supplemental Table S1), suggesting that most of Octopus’ performance advantage comes from better genotyping rather than filtering.

Comparison based on allele matching showed less performance differential between callers, ploidies, and depths, particularly for precision (Supplemental Fig. S7; Supplemental Table S1). However, predominantly because of better sensitivity at low depths, Octopus still made considerably fewer errors in total than GATK (16% fewer) and FreeBayes (36% fewer).

### Longer haplotypes improve genotyping accuracy

**Figure 2.**
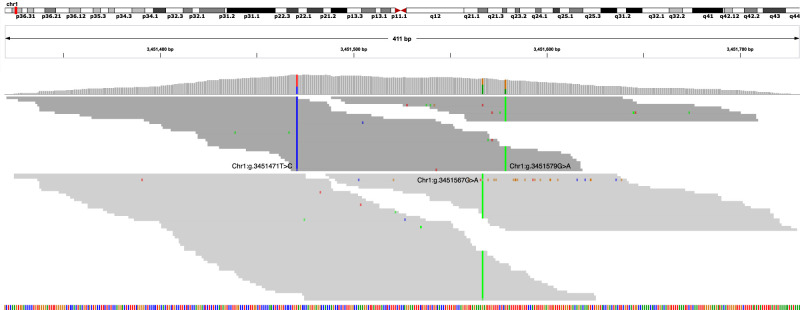
Read pileup of HG003-HG004 tetraploid colored and grouped by supported haplotype. There are two distinct haplotypes (light and dark gray). The true genotypes for the three SNVs (Chr 1:g.3451471T > C, Chr 1:g.3451567G > A, Chr 1:g.3451579G > A) are AAAa, Aaaa, and AAAa. The variant allele read depths are 30/78 (38%), 38/64 (59%), and 20/58 (34%), respectively. GATK4 and FreeBayes both miscall the first two SNVs as AAaa, the most likely genotypes assuming binomially distributed allele observations. Octopus makes the correct calls because it phases all three SNVs, and the first haplotype (including the first and third SNVs) is supported by 74/114 (65%) of reads.

A possible explanation for Octopus having better genotyping accuracy than GATK4 and FreeBayes is that Octopus considered longer haplotypes—on average—when calculating genotype likelihoods. If the true set of haplotypes including a subset of variants can be confidently determined, then the variance in the genotype posterior probability distribution is expected to decrease, with respect to allele-specific copy number, for larger subsets (and therefore longer haplotypes) because the number of discriminating reads is expected to be proportional to the haplotype length (Fig. 2). To test this, we recalled genotypes in the 30× tetraploid sample using a parametrization of Octopus designed to generate longer haplotypes than with default settings (Methods). The mean called haplotype length increased from 319 bases to 511 bases and the number of raw false positives decreased by 3976, but the number of raw false negatives increased by 3117.

### Banana genotyping

Dwarf Cavendish banana (*Musa acuminata*) is autotriploid, consisting of 11 chromosomes with a haploid genome size of ∼523 Mb and is an important food source and export-product for many developing countries (D'Hont et al. 2012). To support our previous results on real polyploid samples, we called variants (Methods) in a Dwarf Cavendish banana specimen that was previously whole-genome-sequenced with two Illumina technologies, NextSeq 500 and HiSeq 1500, to 65× and 55× coverage, respectively (Busche et al. 2020). Both data sets were mapped to the DH Pahang v4 reference (D'Hont et al. 2012; Belser et al. 2021) with BWA-MEM, and genotypes were called with Octopus, GATK4, and FreeBayes.

**Figure 3.**
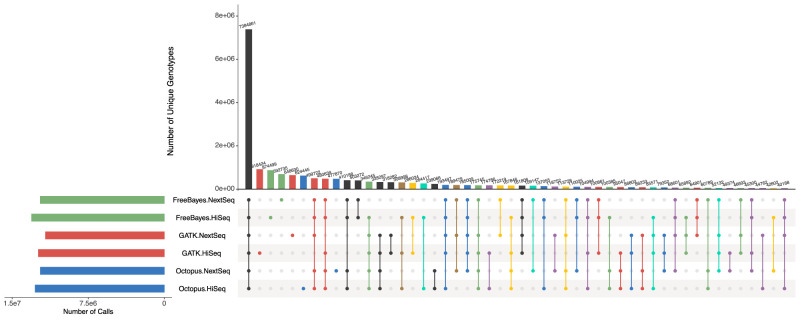
Comparison of genotypes called in two Illumina data sets (HiSeq and NextSeq) of banana specimen by Octopus, GATK4, and FreeBayes. UpSet plot shows callset intersections for each caller–data set pair. The largest 50/63 intersection sets are shown. Intersections are color-coded by caller discordance between the two data sets: no discordances (black), Octopus (blue), GATK4 (red), FreeBayes (green), Octopus & GATK4 (purple), Octopus & FreeBayes (cyan), GATK4 & FreeBayes (yellow), and all (brown). The total number of unique genotype calls was 19,197,247.

Because of the lack of truth data, we evaluated concordance on the two banana data sets using haplotype-aware intersections (Methods). Genotypes called by all callers in both data sets, although substantially the largest intersection set, only accounted for 38% of all distinct genotype calls; 22% of calls were unique to a single callset (Fig. 3). However, there were considerable differences in concordance between the two data sets for each caller: GATK4 had 31% more discordant calls compared with Octopus and had 11% more than FreeBayes, despite making 2.4% fewer calls overall than FreeBayes and only 1% more than Octopus (Table 1). We also found high discordance when intersecting by alleles (Table 1; Supplemental Fig. S8); only 55% of distinct alleles were present in all callsets, whereas 13% were unique to a single callset, indicating that, in comparison to our results on synthetic data, a larger proportion of false calls arise from incorrect variant alleles rather than genotype errors.

**Table 1.**
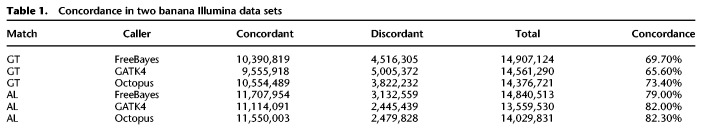
Concordance in two banana Illumina data sets

## Discussion

We have shown that genotyping is substantially more error-prone in polyploids than in diploids using typical whole-genome sequencing depths, emphasizing that polyploid sequencing studies must be carefully designed to ensure sufficient sequencing depth and caution taken when interpreting polyploid genotype calls. We also found considerable differences in accuracy between callers. Octopus produced fewer than a quarter of the total errors of other methods and half the errors on some data sets. We believe this may be owing to Octopus modeling longer haplotypes, on average, as at loci where variant phasing can be confidently determined, reads assignable to any one allele on a haplotype are implicitly assigned to all other alleles on the same haplotype—even if the read does not overlap them—therefore increasing the effective allele-specific observation count. Regenotyping with longer haplotypes increased genotyping precision, supporting this hypothesis.

Analysis of real autotriploid banana data sets revealed high discordance between callers, as well as high discordance for all callers on a technical replicate. Although these results were at least consistent with the relative accuracy of callers determined by our benchmarks using synthetic polyploid data (Octopus was the most concordant caller), absolute error rates were evidently higher in real polyploid data. Reasons for this may include greater divergence from the reference genome (Busche et al. 2020); higher levels of repetitive elements in the genome (Jackson et al. 2011); more structural variation (Busche et al. 2020); a less complete reference genome (Jackson et al. 2011); higher rates of sequencing related errors, such as owing to the use of PCR amplification; and bioinformatics algorithms optimized for human data.

We have only considered single-sample polyploid calling in this work; however, multisample calling is important for studying population diversity. Population calling in humans is a difficult problem owing to the computational complexities of joint calling and difficulties in merging independent callsets. Population calling in polyploids will likely be even more challenging and would perhaps benefit from more sophisticated genotype prior models (Blischak et al. 2018).

Moving forward, there is clearly room for improvement in polyploid genotyping from sequencing. The creation of high-quality validation sets with real polyploid samples would be highly valuable in the development of polyploid-calling algorithms, including Octopus. We hope that this work lays the groundwork for future developments.

## Methods

### Synthetic polyploids with real reads

Raw reads (FASTQ) generated for the PrecisionFDA Truth v2 challenge (Olson et al. 2021) were downloaded from the DNAnexus portal (https://precision.fda.gov/challenges/10). Each FASTQ was line-counted to ensure realistic haplotype frequencies, before concatenation of contributing samples to make the full data polyploid data set. Down-sampling was performed directly on the FASTQ files using *seqtk* with the default seed. The sampling fraction was set using *test depth*/*full depth*, where full depth is 35× *ploidy*/2. Reads were mapped with BWA-MEM using default alignment parameters.

### Creating polyploid truth genotypes from diploid GIAB samples

Polyploid truth genotypes were generated by concatenating diploid genotypes from GIAB truth VCFs using the BCFtools *merge* and the RTG Tools *vcffilter*, *vcfannotate*, and *vcfsubset* commands. We note that this merge procedure does not resolve variant representation differences between samples. High-confidence BED regions were generated by intersecting GIAB high-confidence BED regions with the BEDTools *multiinter*.

### Changes to Octopus for polyploid calling

Although the models that we previously described for Octopus (Cooke et al. 2021) are fully capable of polyploid calls, in practice we found some issues. Runtimes were prohibitive for high ploidies owing to the model always considering every possible genotype for a given set of candidate haplotypes, which is reasonable for diploids but not polyploids. Moreover, sensitivity for simplex variants was not optimal owing to the variant discovery mechanisms not fully accounting for ploidy.

To resolve the runtime issue, we modified the genotype proposal algorithm so that an upper bound on the number of genotypes evaluated can be specified. The algorithm respects this limit by evaluating the full model on the maximum ploidy that results in fewer candidate genotypes than the limit for a given set of haplotypes and then extends a subset of these with greatest posterior probability using each of the candidate haplotypes. The procedure is then applied iteratively, increasing the ploidy by one each iteration until the desired ploidy is reached. We expect this procedure to work well when the number of unique haplotypes present in a region is not substantially greater than the first ploidy considered. We addressed the sensitivity issue by tweaking the pileup and local de novo reassembly candidate variant discovery algorithms to account for the sample ploidy. Octopus source code and documentation are freely available under the MIT license from GitHub (https://github.com/luntergroup/octopus).

### Variant calling polyploids

For GATK4, we called variants using BAMs with marked duplicates created by GATK4's *MarkDuplicates* tool. Raw BAMs were used for FreeBayes and Octopus. The sample ploidy was specified for all callers: ‐‐*organism-ploidy* (Octopus), ‐‐*sample-ploidy* (GATK4), and ‐‐*ploidy* (FreeBayes). For FreeBayes, we requested genotype qualities with the - = option.

### Filtering variant calls

For GATK4, we used following filter expressions: “-*filter ‘QD < 2.0’ ‐‐filter-name ‘QD2’ -filter ‘QUAL < 50’ ‐‐filter-name ‘Q50’ -filter ‘GQ < 5’ ‐‐filter-name ‘GQ5’ -filter ‘FS > 60.0’ ‐‐filter-name ‘FS60’ -filter ‘SOR > 3.0’ ‐‐filter-name ‘SOR3’ -filter ‘MQ < 40.0’ ‐‐filter-name ‘MQ40’ -filter ‘MQRankSum < −12.5’ ‐‐filter-name ‘MQRankSum-12.5’ -filter ‘ReadPosRankSum < −8.0’ ‐‐filter-name ‘ReadPosRankSum-8*.’” For FreeBayes, we used filter expression “*QUAL > 1 & GQ > 1 & SAF > 0 & SAR > 0*.”

### Genotype and allele comparisons

We used RTG Tools vcfeval (v3.12.1) for genotype and allele comparisons, using the ‐‐*sample-ploidy* and ‐‐*ref-overlap* options. For allele matching, we also used the ‐‐*squash-ploidy*, ‐‐*XXcom.rtg.vcf.eval.flag-alternates = true*, and ‐‐*output-mode = “annotate”* options and then determined true and false calls based on the resulting BASE, CALL, BASE_ALTERNATE, and CALL_ALTERNATE annotations.

### Identifying genotype errors

Biallelic genotype errors were identified by running RTG Tools vcfeval with the ‐‐*output-mode = “combine”* option and considering biallelic calls with baseline INFO annotations “BASE = FN_CA” and “CALL = FP_CA.”

### Long haplotypes with Octopus

To call long haplotypes with Octopus, we provided Octopus with the variant calls it previously produced with default setting as candidates (‐‐*source-candidates*) and disabled de novo variant discovery (‐‐*disable-denovo-variant-discovery*). We also set command line options ‐‐*lagging-level = OPTIMSITIC*, ‐‐*backtrack-level = AGGRESSIVE*, and ‐‐*max-haplotypes = 400*.

### Banana concordance analysis

Callsets for the banana data sets were intersected using a custom script (https://github.com/dancooke/starfish) that invokes both RTG Tools vcfeval (that only supports two-way comparisons) and BCFtools to achieve multisample haplotype-aware comparisons. UpSet plots were created with UpSetR (Conway et al. 2017).

### Software availability

Custom Snakemake (Köster and Rahmann 2018) and Python code used for data analysis are available from GitHub (https://github.com/luntergroup/polyploid) and as Supplemental Code.

## Supplementary Material

Supplemental Material
