# Supplementary material for: Benchmarking small-variant genotyping in polyploids
Source: Genome Res. 2022 Feb;32(2):403–8. doi: 10.1101/gr.275579.121 (PMC8805713; doi:10.1101/gr.275579.121)
Supplement: Supplemental Material [file supp_32_2_403__DC1.html]

Benchmarking small-variant genotyping in polyploids — Supplemental Material 

# Benchmarking small-variant genotyping in polyploids

## Supplemental Material

- Supplemental\_Code.zip
- Supplemental\_Material.pdf
- Supplemental\_Table\_S1.xlsx
- Supplemental\_Table\_S2.xlsx
- Supplemental\_Table\_S3.xlsx
- Supplemental\_Table\_S4.xlsx
