# Supplementary material for: Benchmarking small-variant genotyping in polyploids
Source: Genome Res. 2022 Feb;32(2):403–8. doi: 10.1101/gr.275579.121 (PMC8805713; doi:10.1101/gr.275579.121)
Supplement: Supplemental Material [file supp_gr.275579.121_Supplemental_Code.zip › polyploid-1.0.0/paper/supplementary/supplementary.pdf]

Benchmarking small-variant genotyping in polyploids: supplementary material

1 Supplementary Tables and Figures

(a)

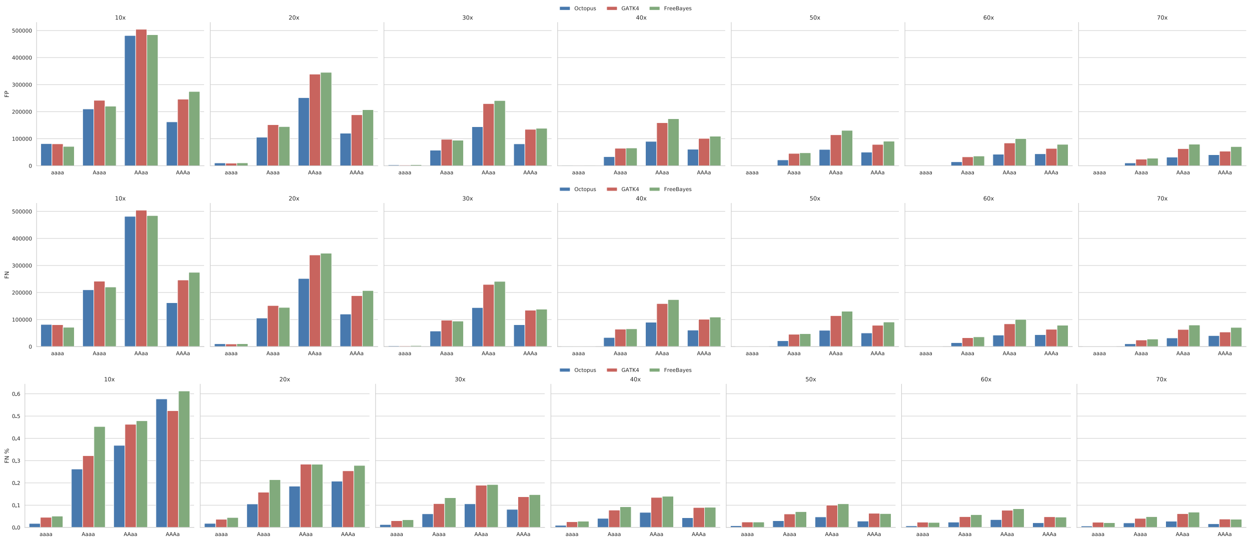

(b)

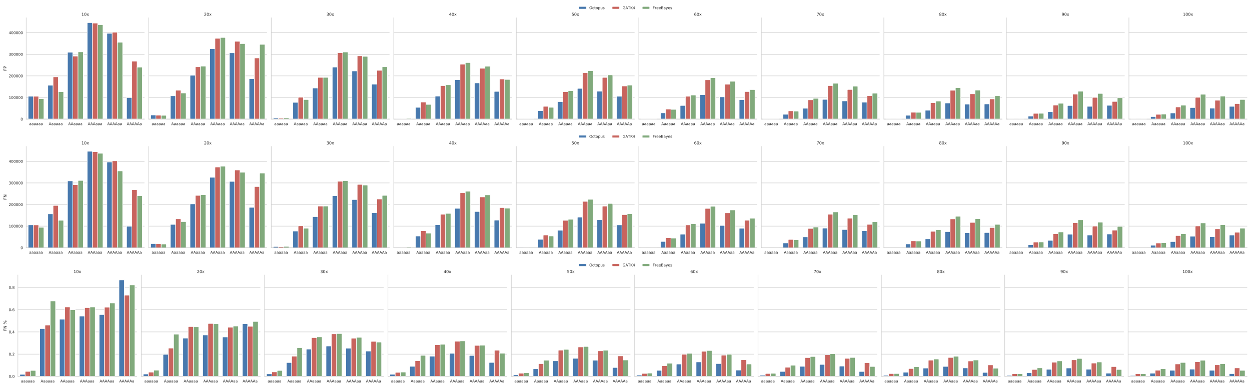

Supplementary Figure. 1 — Biallelic genotyping errors in synthetic polyploid samples. a Tetraploid. **b** Hexaploid.

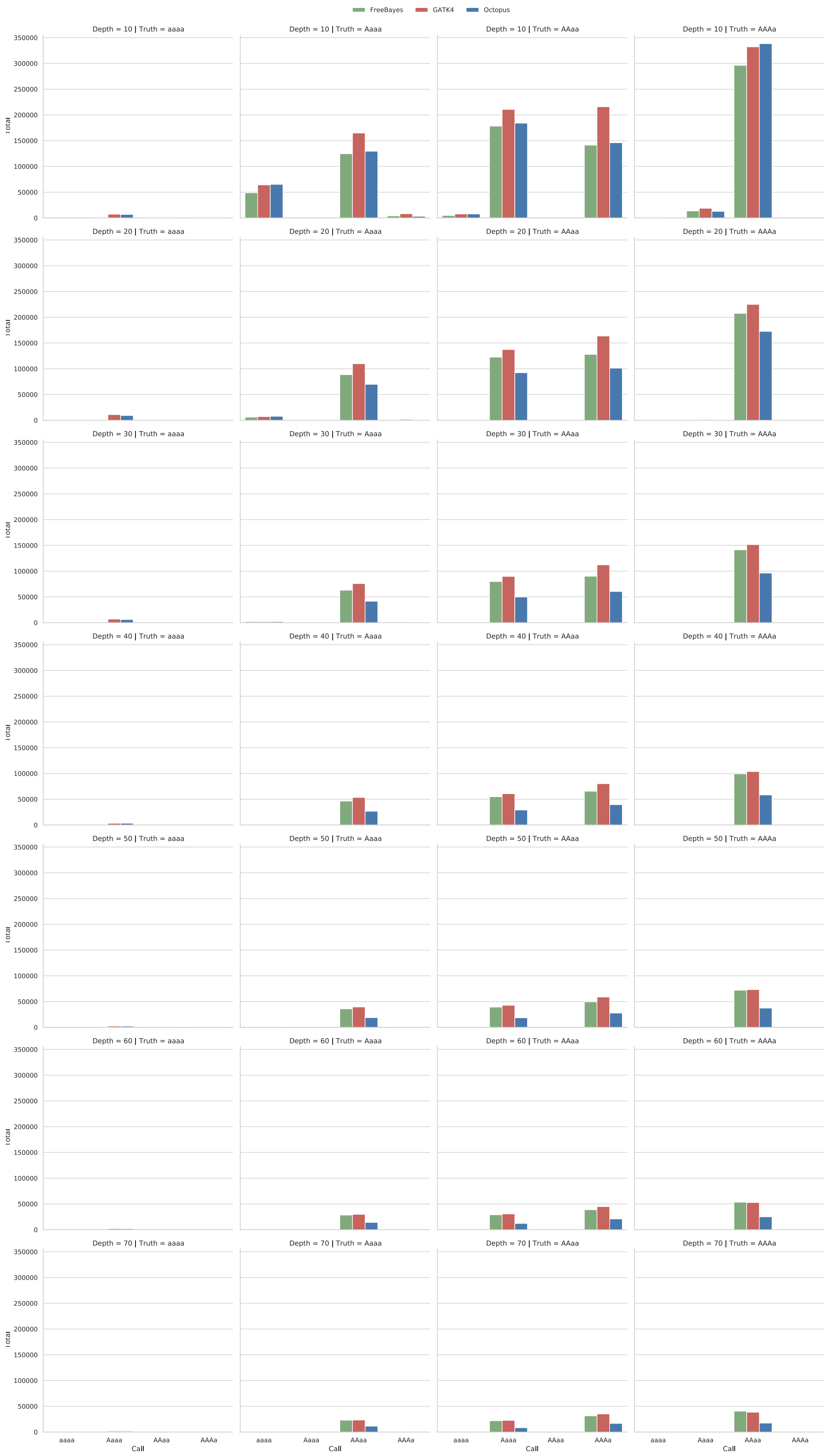

Supplementary Figure. 2 — Biallelic copy number errors in synthetic tetraploid samples.

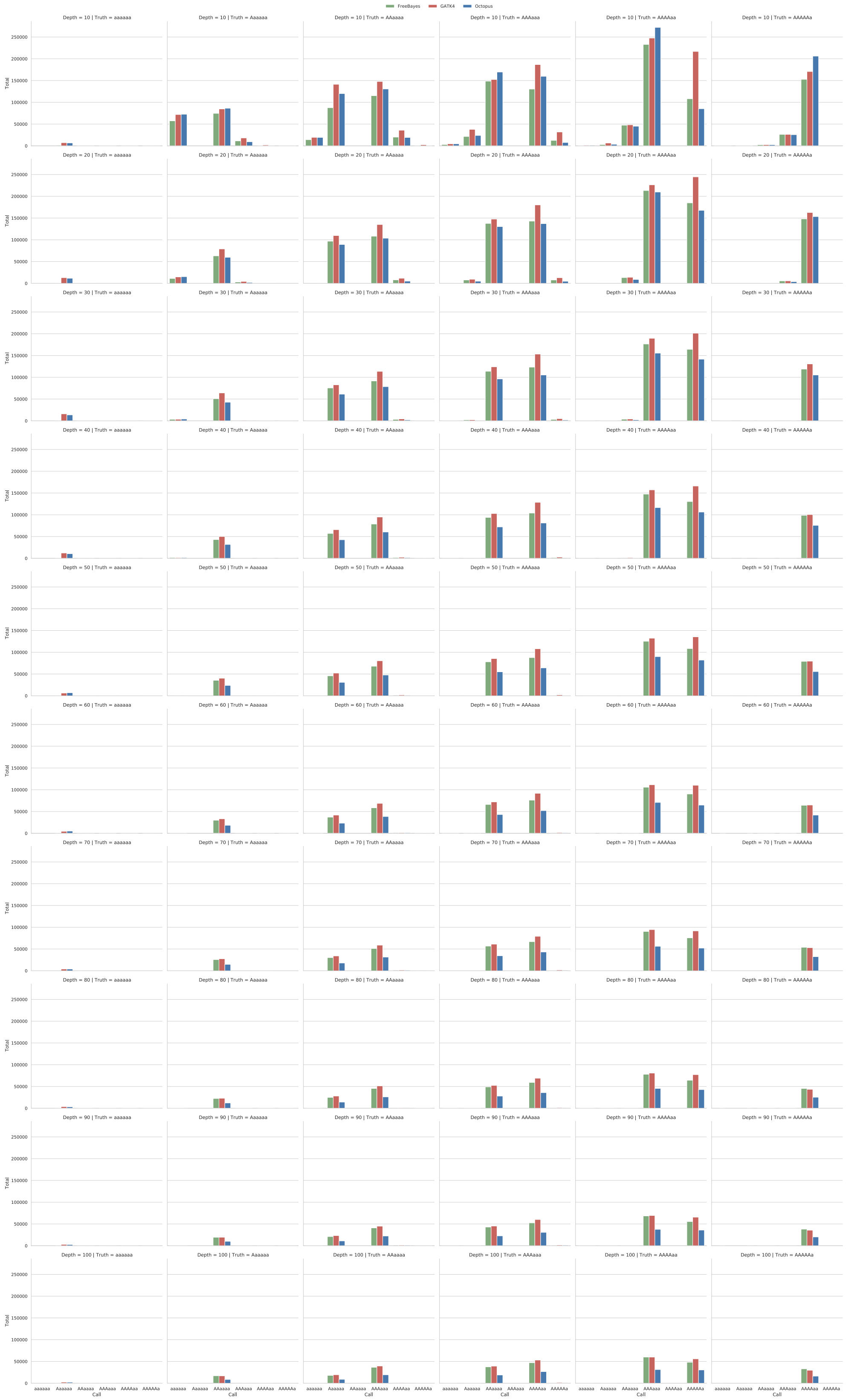

Supplementary Figure. 3 — Biallelic copy number errors in synthetic hexaploid samples.

(a)

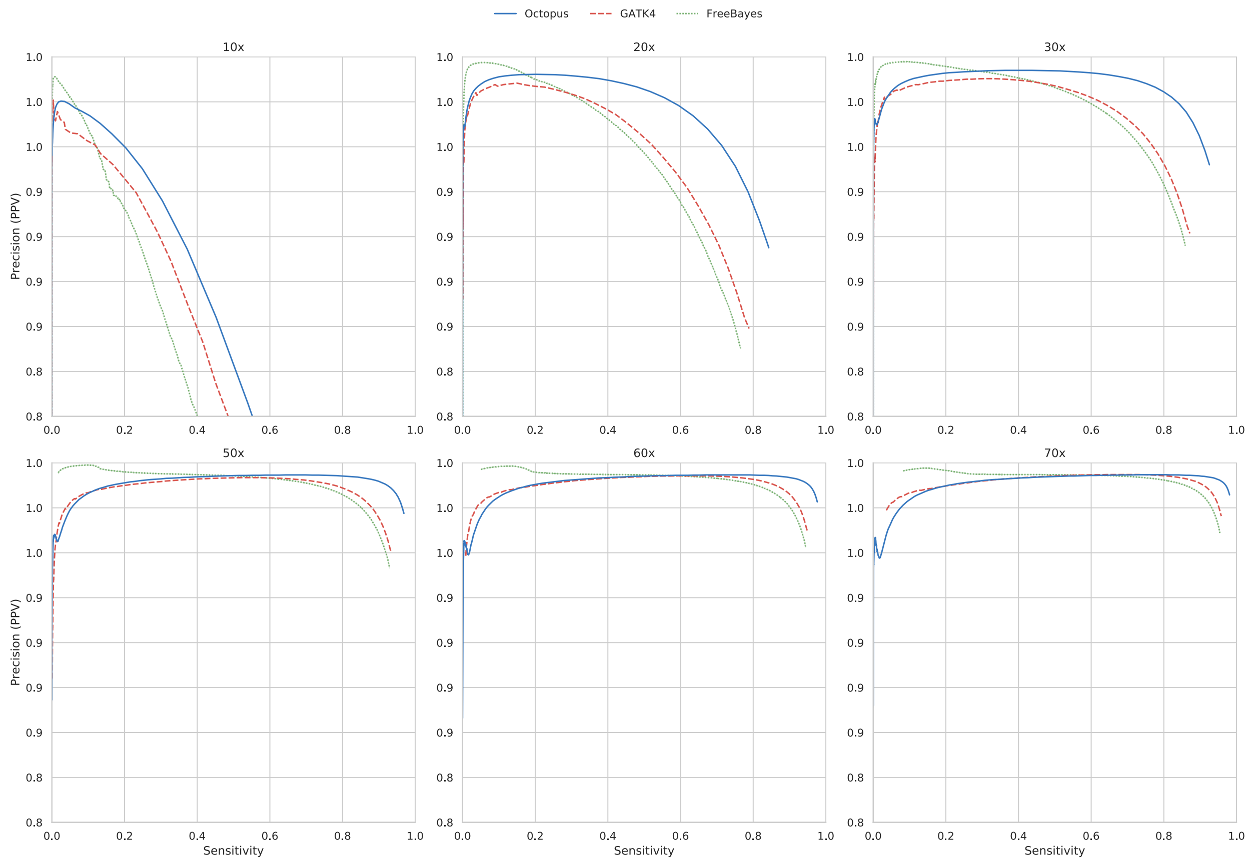

(b)

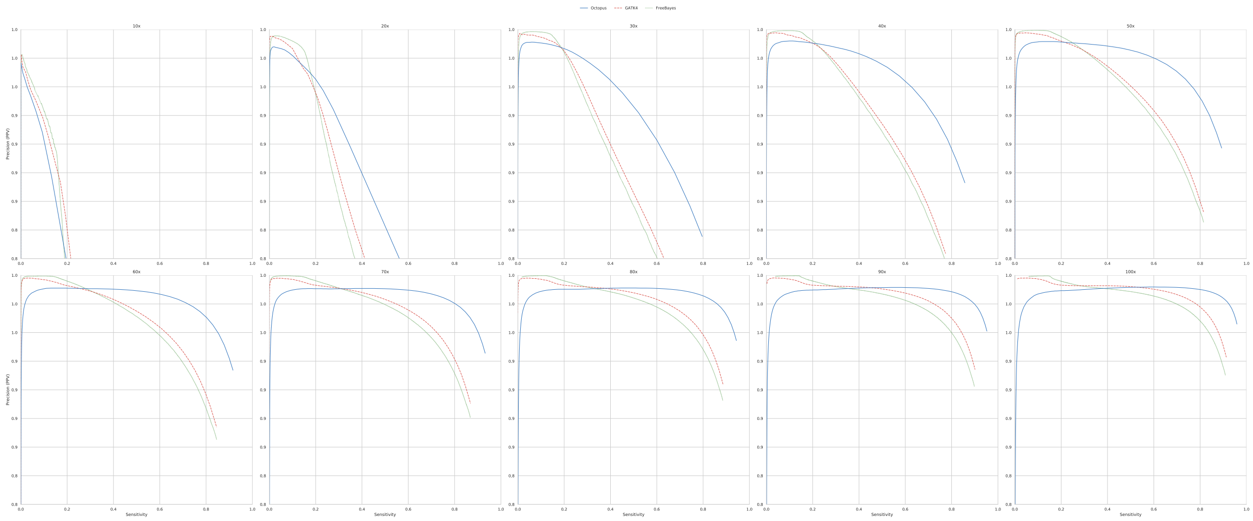

Supplementary Figure. 4 — Precision-recall curves for genotyping errors in synthetic polyploid samples. GQ was used to generate curves for all samples. **a** Tetraploid. **b** Hexaploid.

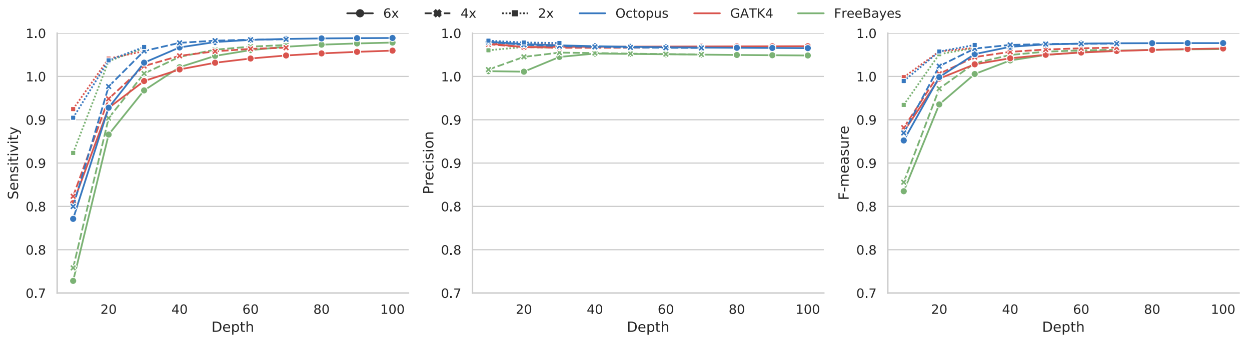

Supplementary Figure. 5 — Comparison of allele calling accuracies for diploid and synthetic polyploid datasets.

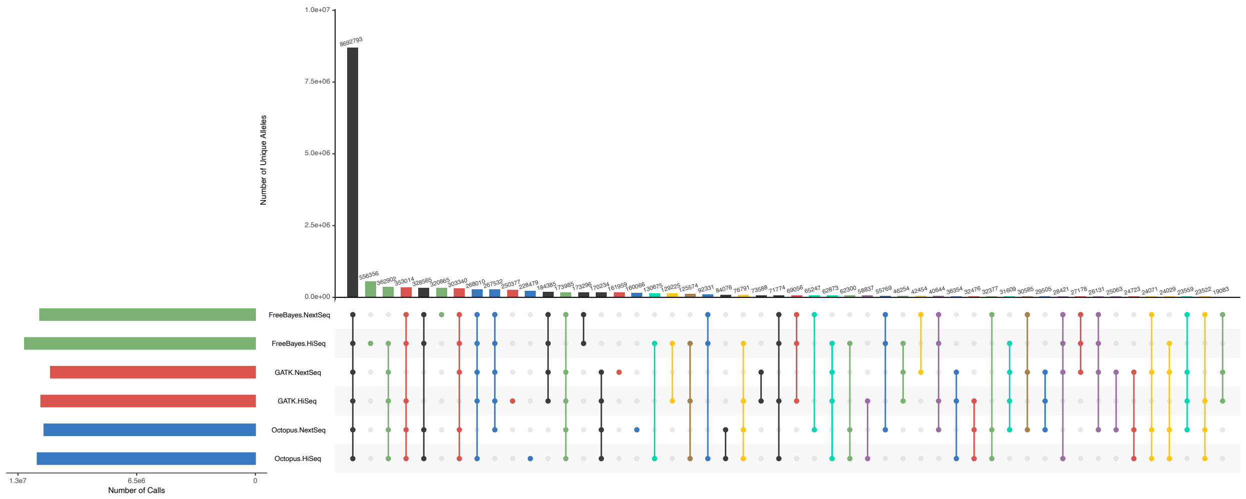

Supplementary Figure. 6 — Comparison of alleles called in two Illumina datasets (HiSeq and NextSeq) of banana specimen by Octopus, GATK4, and FreeBayes. UpSet plot shows callset intersections for each caller-dataset pair. The largest 50/63 intersection sets are shown. Intersections are color coded by caller discordance between the two datasets: No discordances (black), Octopus (blue), GATK4 (red), FreeBayes (green), Octopus & GATK4 (purple), Octopus & FreeBayes (cyan), GATK4 & FreeBayes (yellow), All (brown). The total number of unique alleles calls was 14,825,250.
