# Supplementary figures and images for: Benchmarking small-variant genotyping in polyploids
Source: Genome Res. 2022 Feb;32(2):403–8. doi: 10.1101/gr.275579.121 (PMC8805713; doi:10.1101/gr.275579.121)

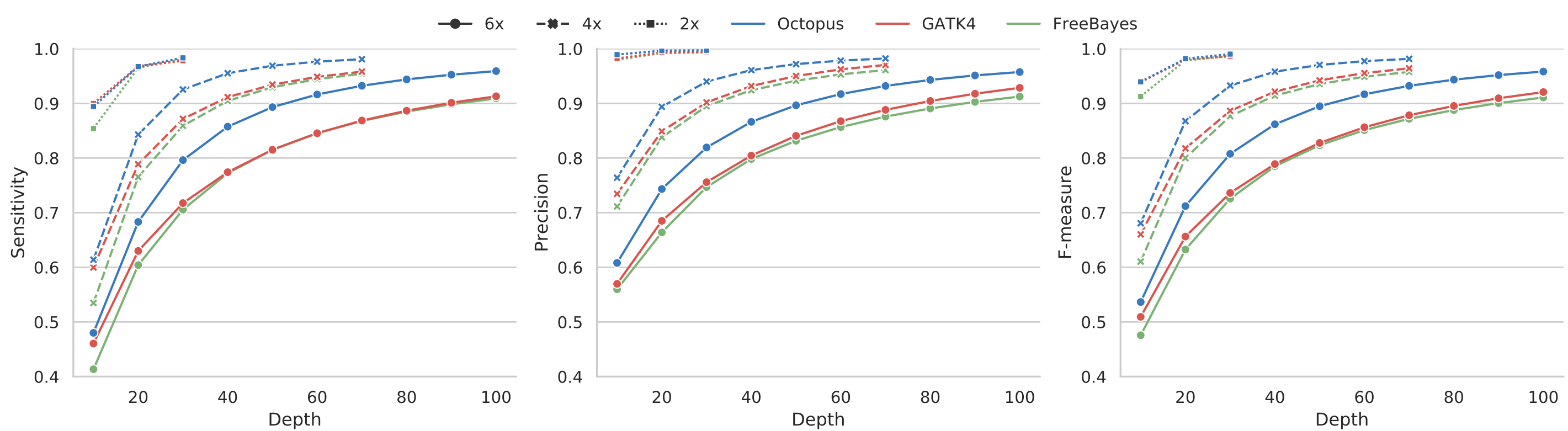

Supplement: Supplemental Material [file supp_gr.275579.121_Supplemental_Code.zip › polyploid-1.0.0/paper/main/figures/accuracies-by-depth.pdf]

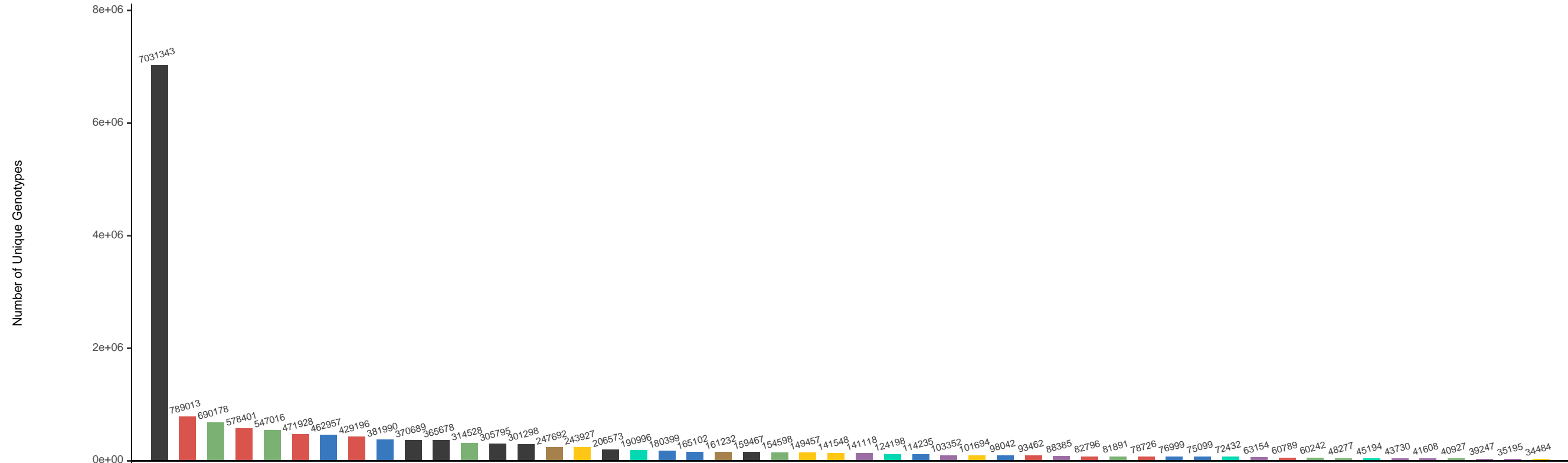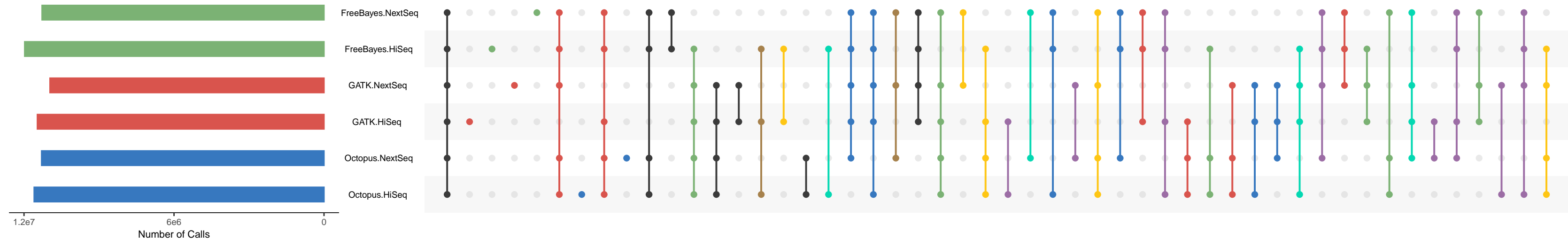

Supplement: Supplemental Material [file supp_gr.275579.121_Supplemental_Code.zip › polyploid-1.0.0/paper/main/figures/banana_intersections.pdf]

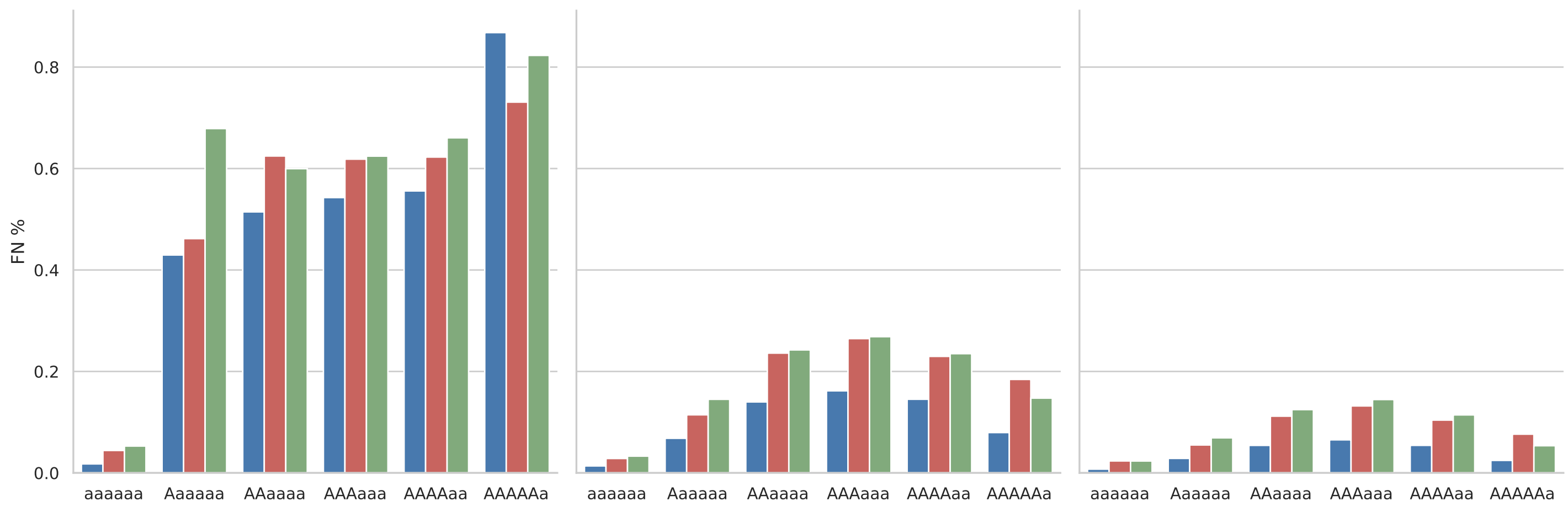

Supplement: Supplemental Material [file supp_gr.275579.121_Supplemental_Code.zip › polyploid-1.0.0/paper/main/figures/hexaploid_gt_fn_perc_10x_50x_100x.pdf]

Octopus GATK4 FreeBayes

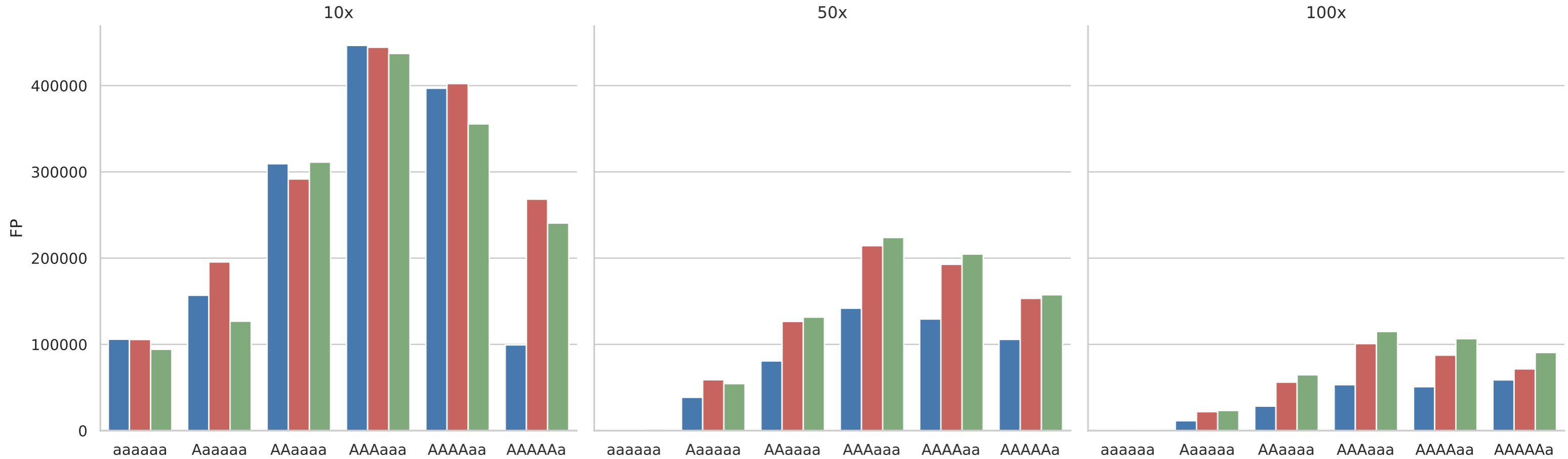

Supplement: Supplemental Material [file supp_gr.275579.121_Supplemental_Code.zip › polyploid-1.0.0/paper/main/figures/hexaploid_gt_fp_10x_50x_100x.pdf]

Octopus GATK4 FreeBayes

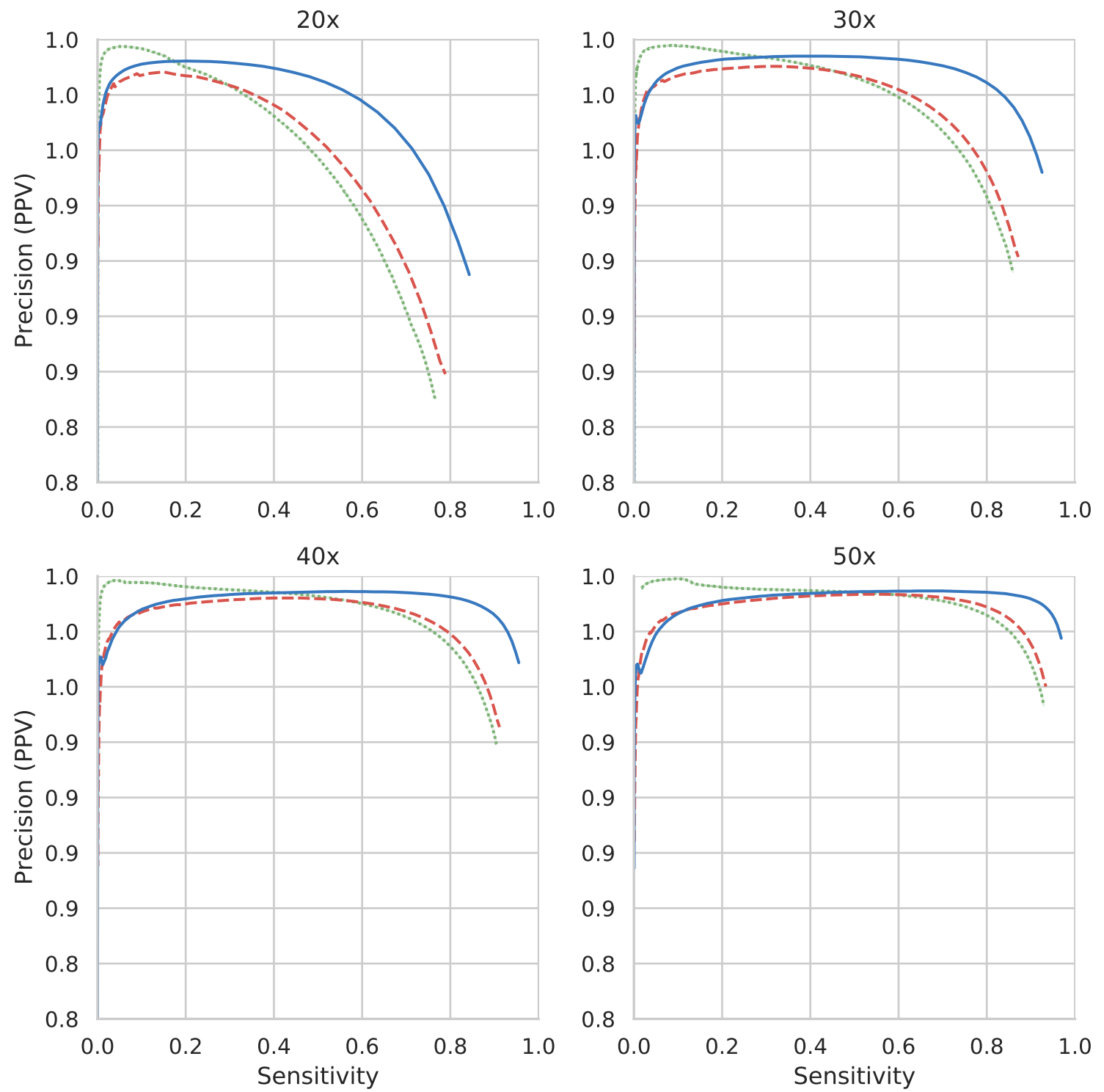

Supplement: Supplemental Material [file supp_gr.275579.121_Supplemental_Code.zip › polyploid-1.0.0/paper/main/figures/synthetic-tetraploid-pr-curves_20-50x.pdf]

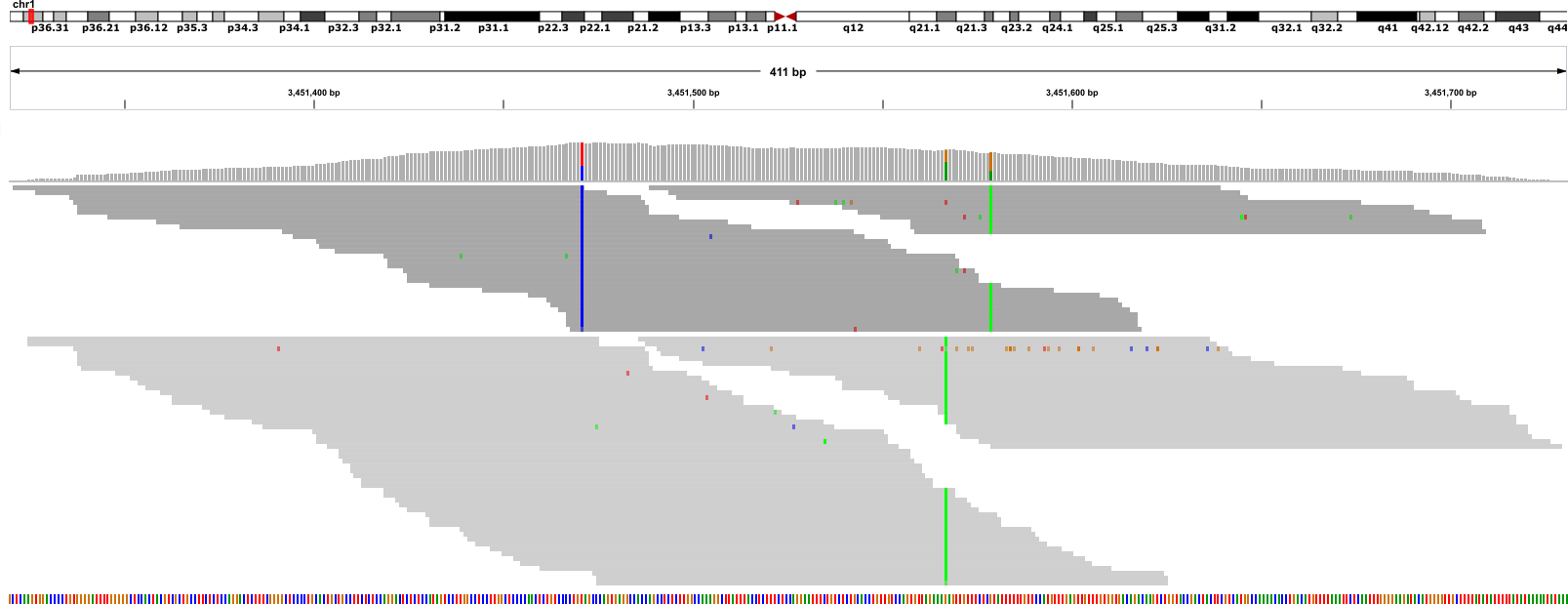

Supplement: Supplemental Material [file supp_gr.275579.121_Supplemental_Code.zip › polyploid-1.0.0/paper/main/figures/tetraploid_haplotypes.png]

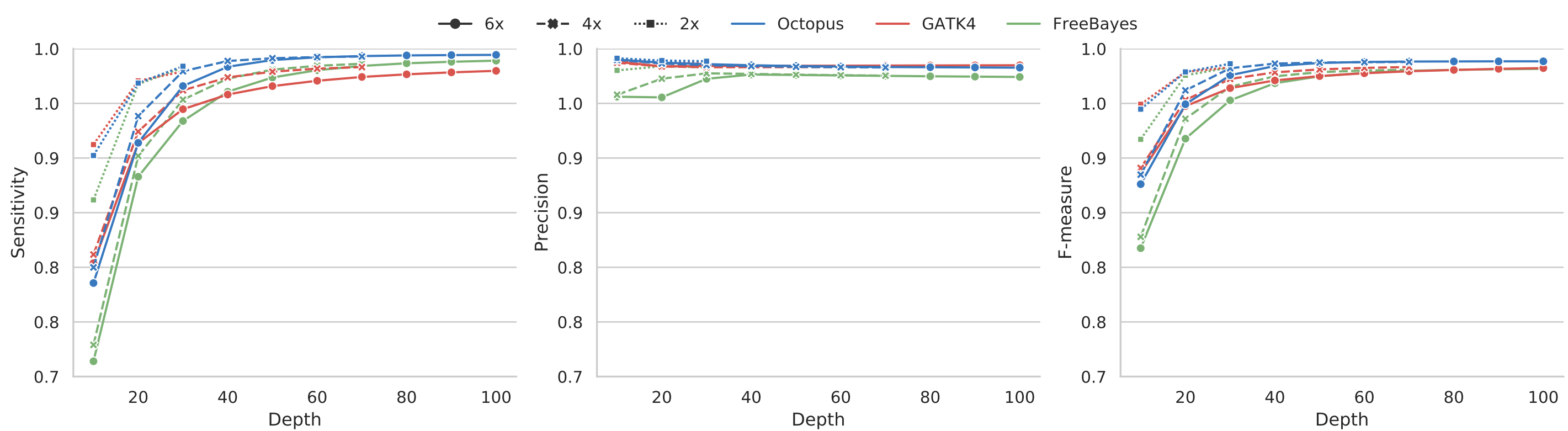

Supplement: Supplemental Material [file supp_gr.275579.121_Supplemental_Code.zip › polyploid-1.0.0/paper/supplementary/figures/accuracies-by-depth-alleles.pdf]

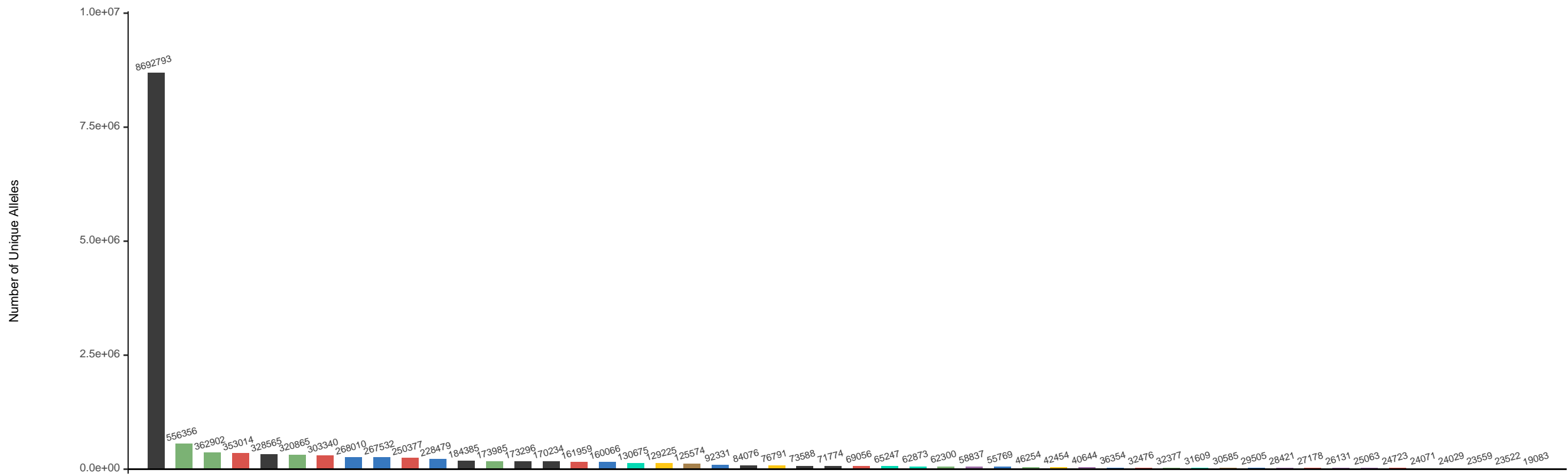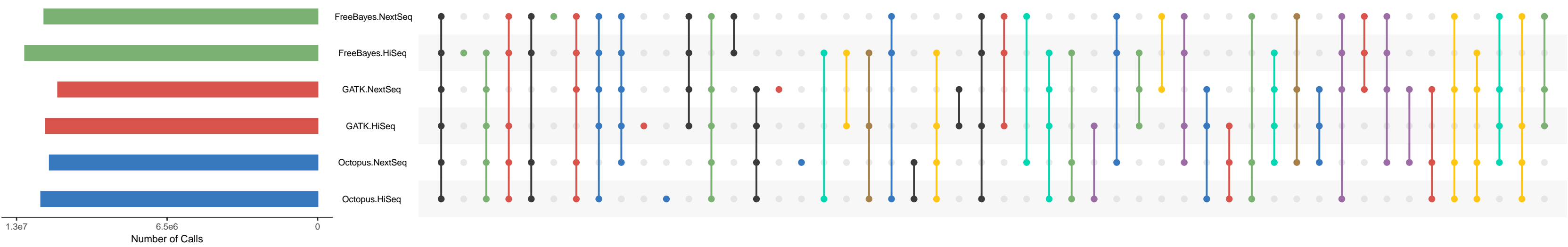

Supplement: Supplemental Material [file supp_gr.275579.121_Supplemental_Code.zip › polyploid-1.0.0/paper/supplementary/figures/banana_intersections_alleles.pdf]

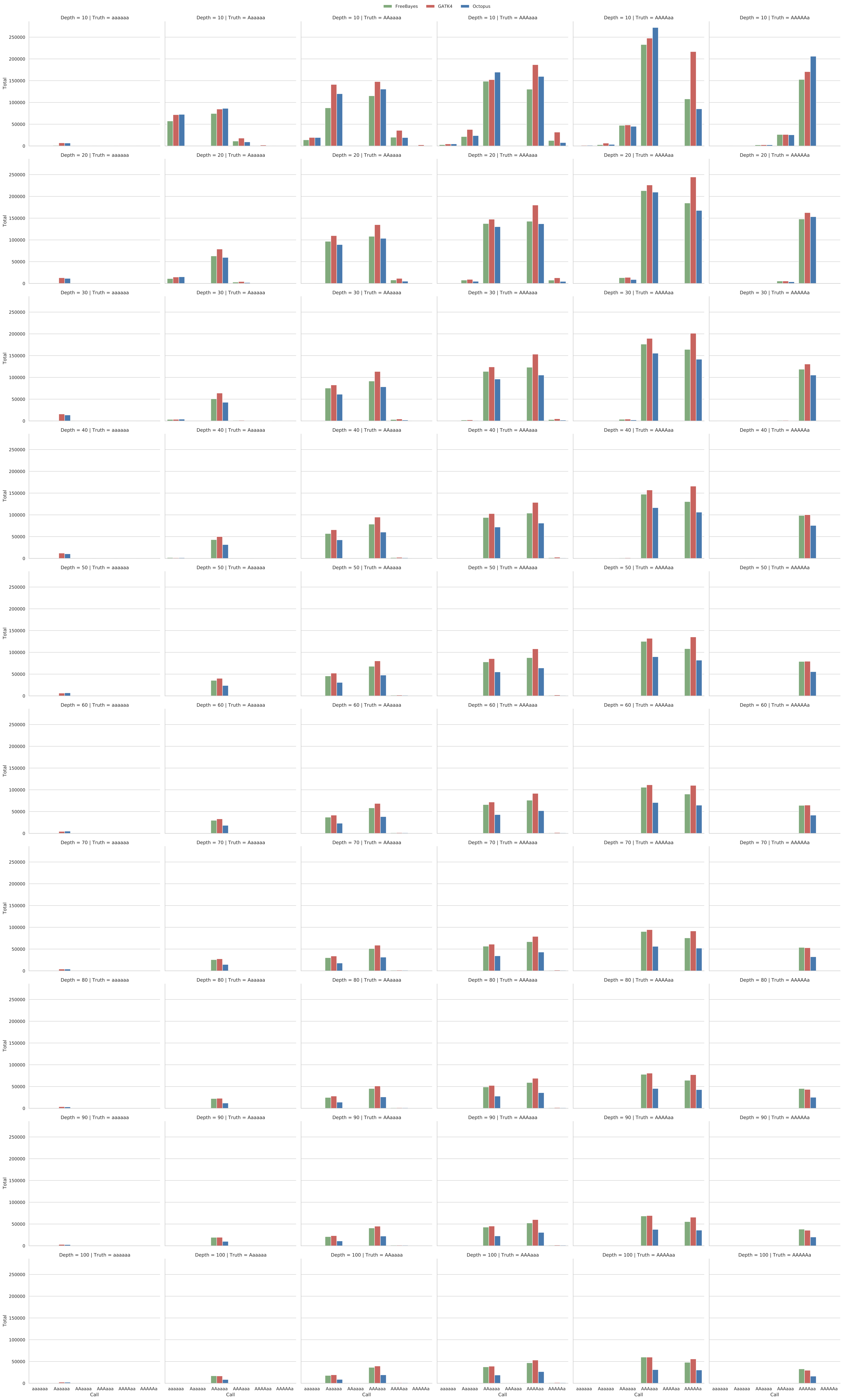

Supplement: Supplemental Material [file supp_gr.275579.121_Supplemental_Code.zip › polyploid-1.0.0/paper/supplementary/figures/hexaploid_biallelic_copy_errors.pdf]

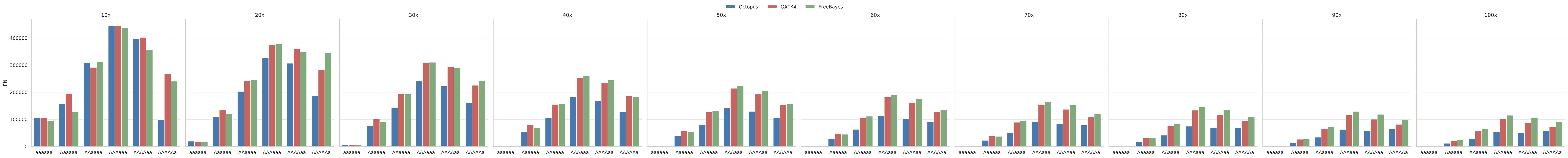

Supplement: Supplemental Material [file supp_gr.275579.121_Supplemental_Code.zip › polyploid-1.0.0/paper/supplementary/figures/hexaploid_gt_fn.pdf]

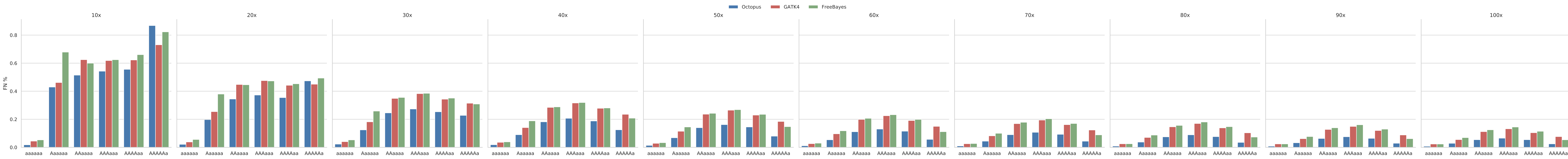

Supplement: Supplemental Material [file supp_gr.275579.121_Supplemental_Code.zip › polyploid-1.0.0/paper/supplementary/figures/hexaploid_gt_fn_perc.pdf]

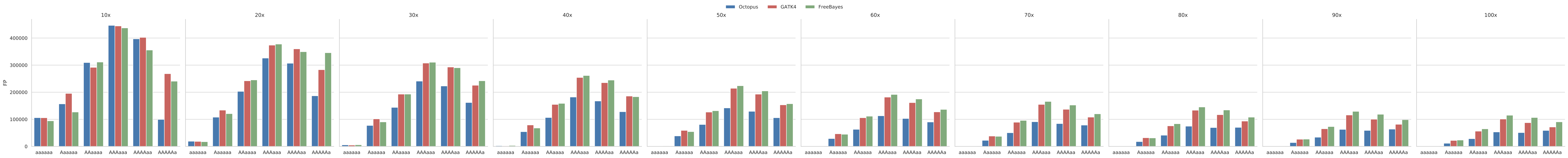

Supplement: Supplemental Material [file supp_gr.275579.121_Supplemental_Code.zip › polyploid-1.0.0/paper/supplementary/figures/hexaploid_gt_fp.pdf]

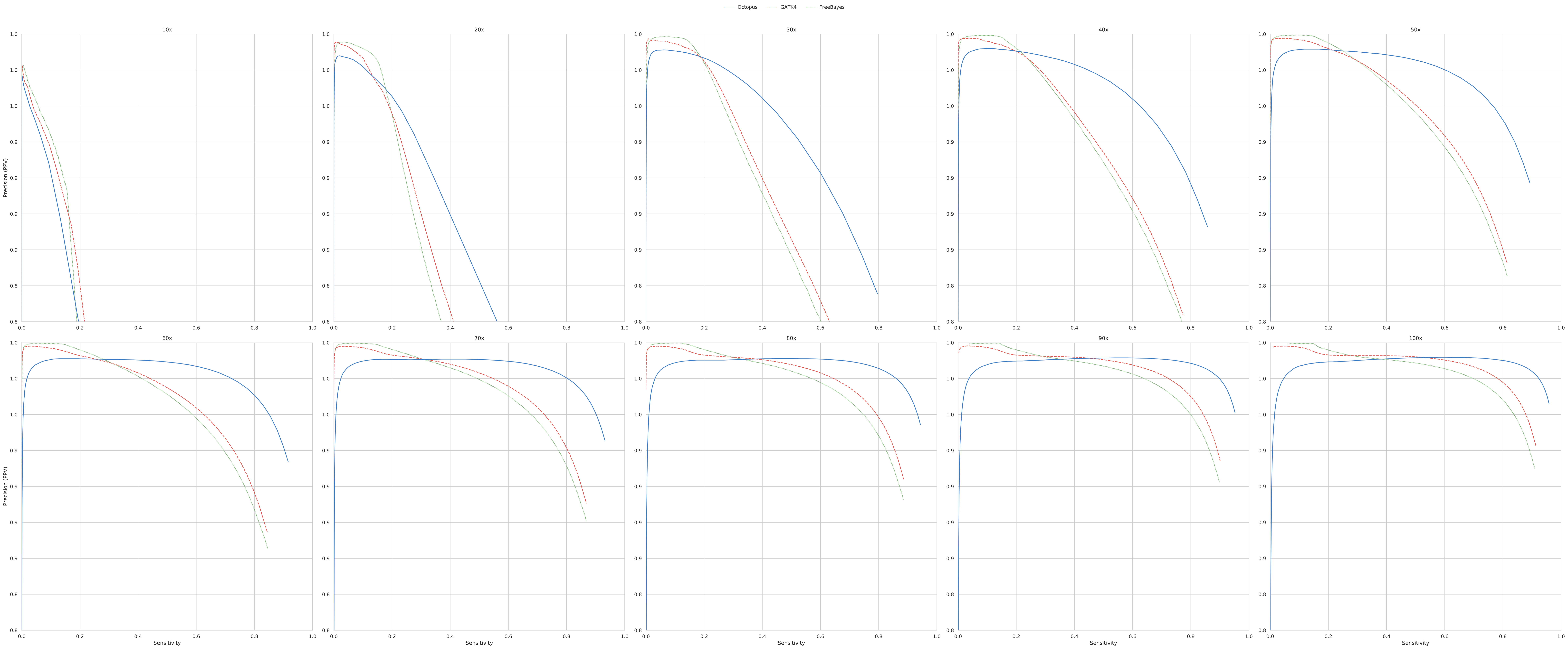

Supplement: Supplemental Material [file supp_gr.275579.121_Supplemental_Code.zip › polyploid-1.0.0/paper/supplementary/figures/synthetic-hexaploid-pr-curves.pdf]

Octopus GATK4 FreeBayes

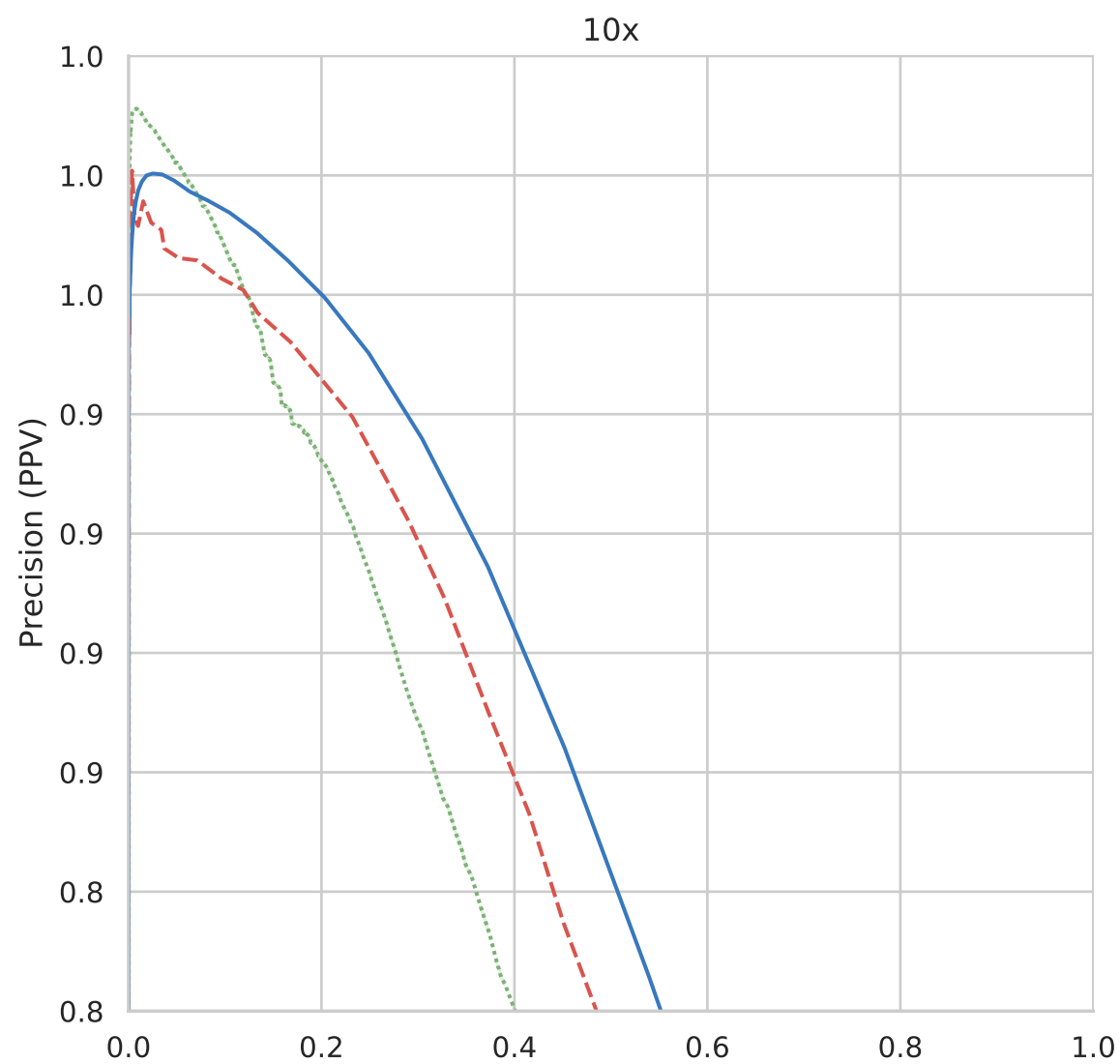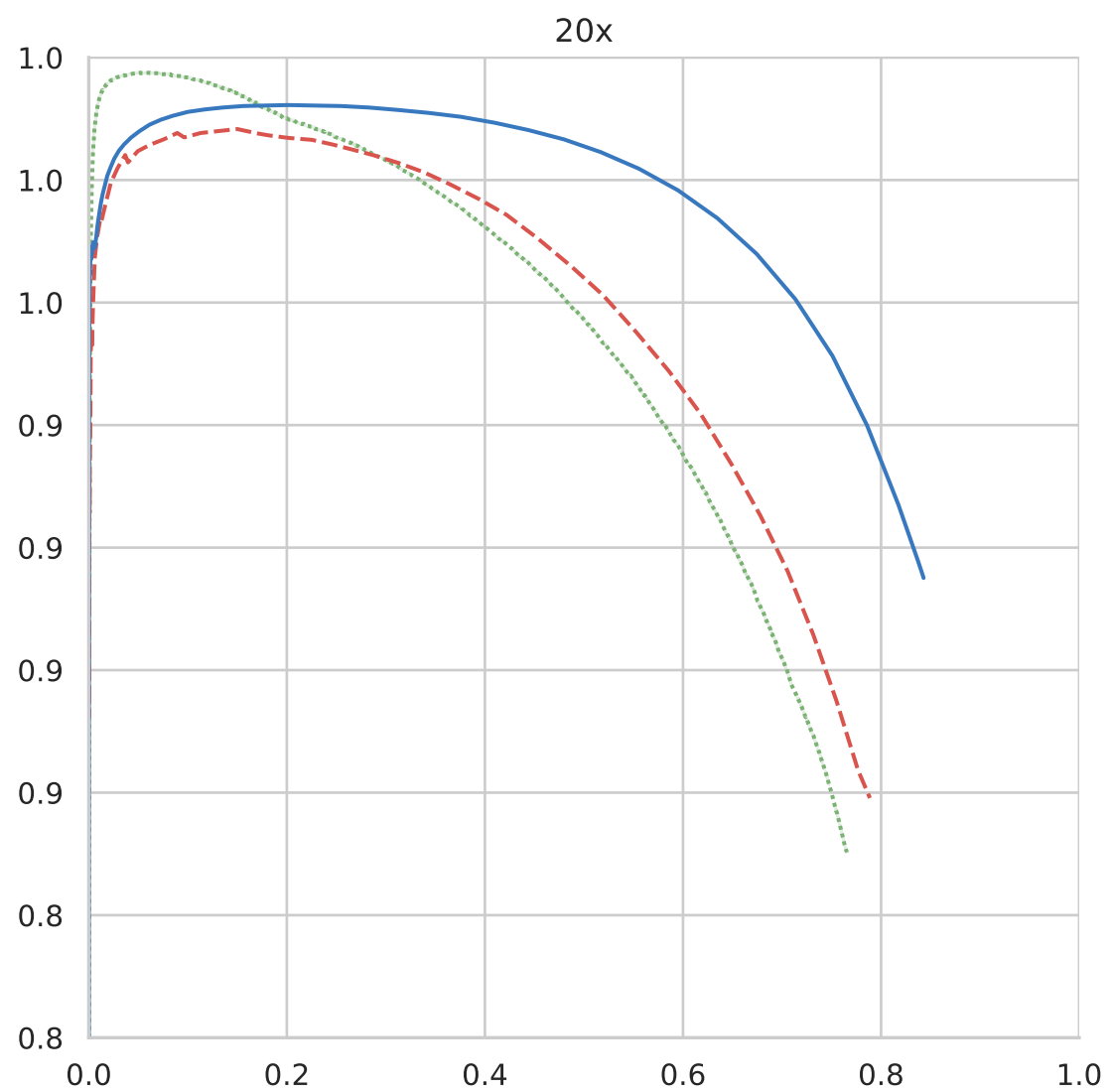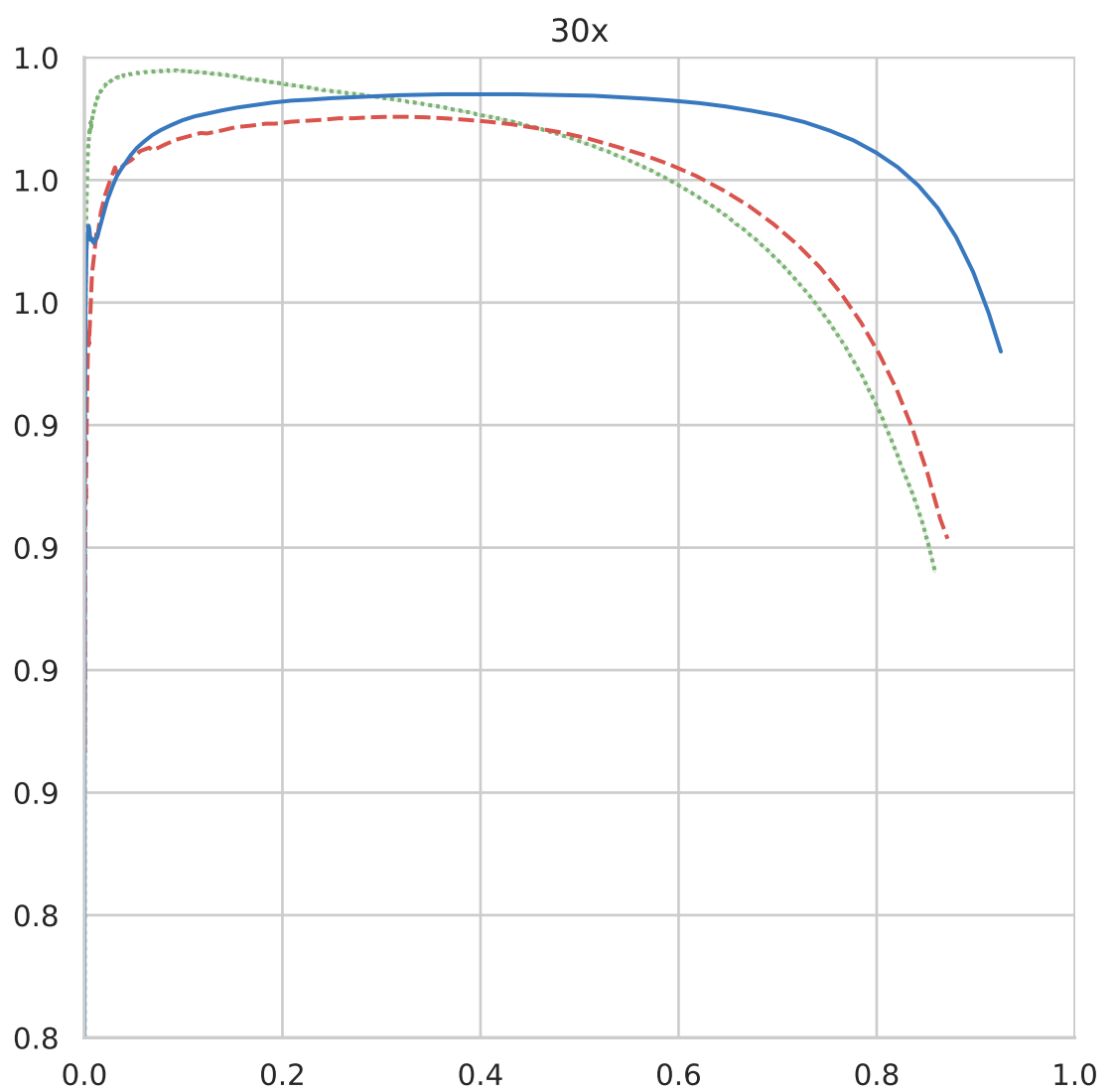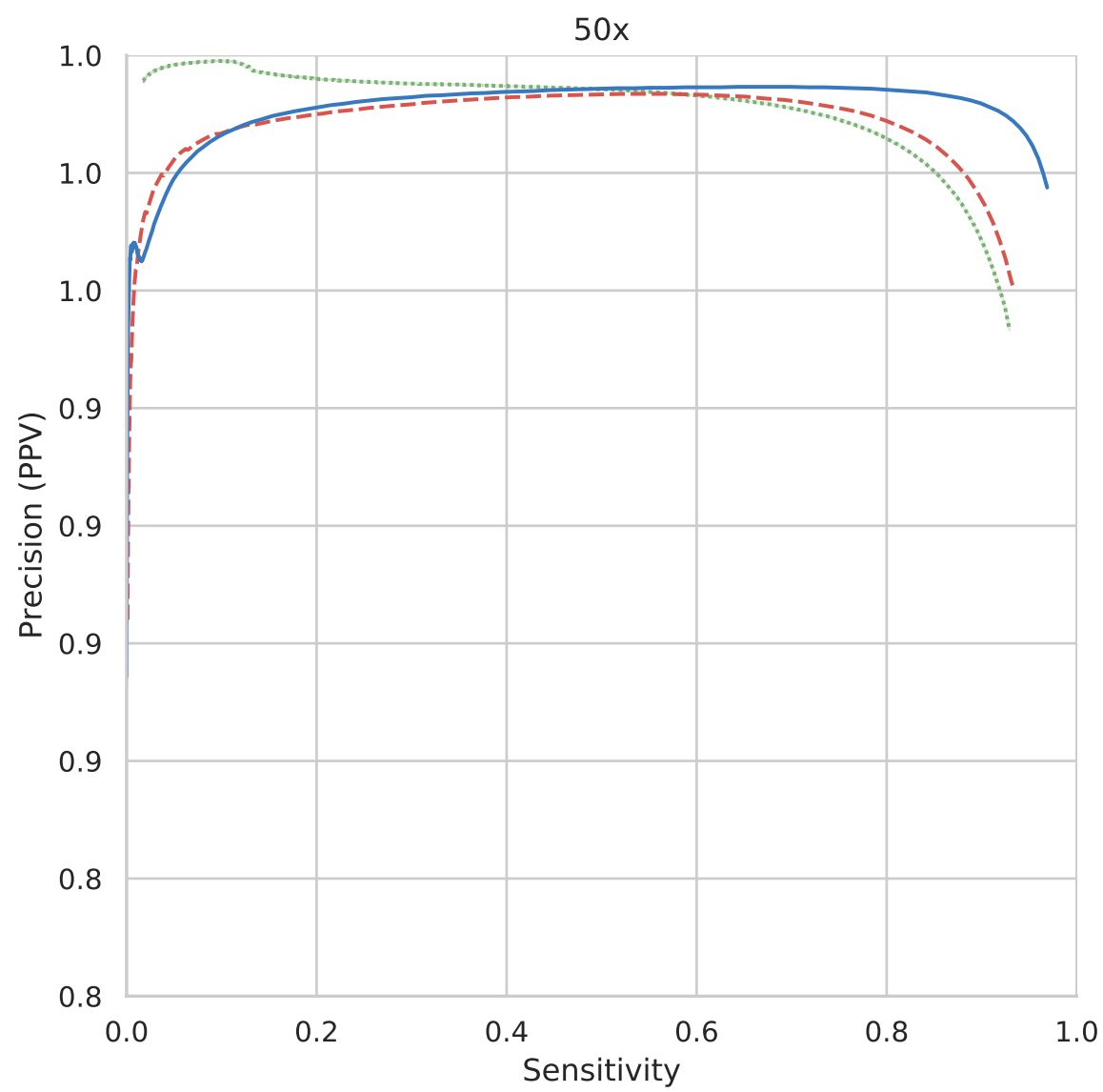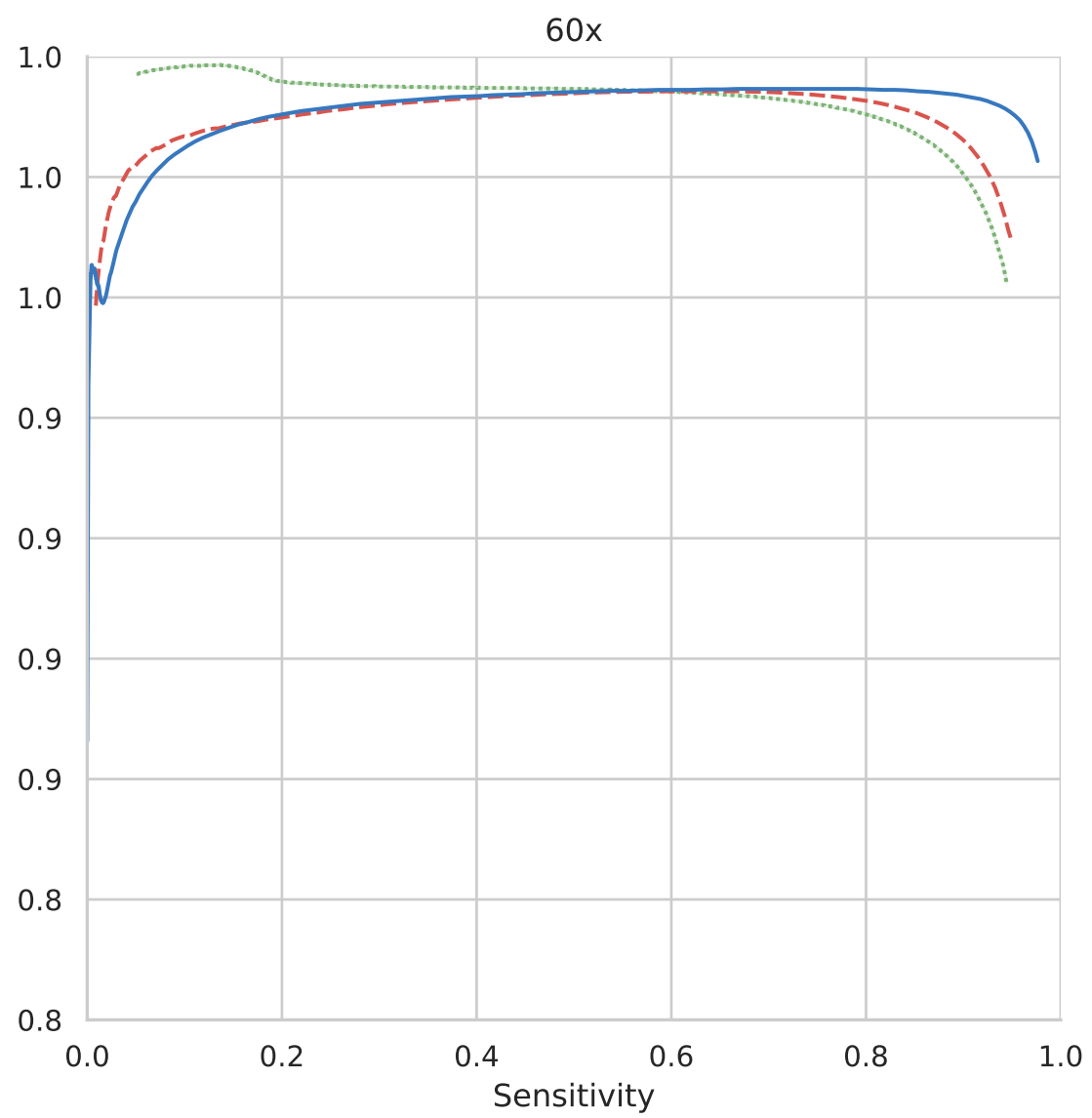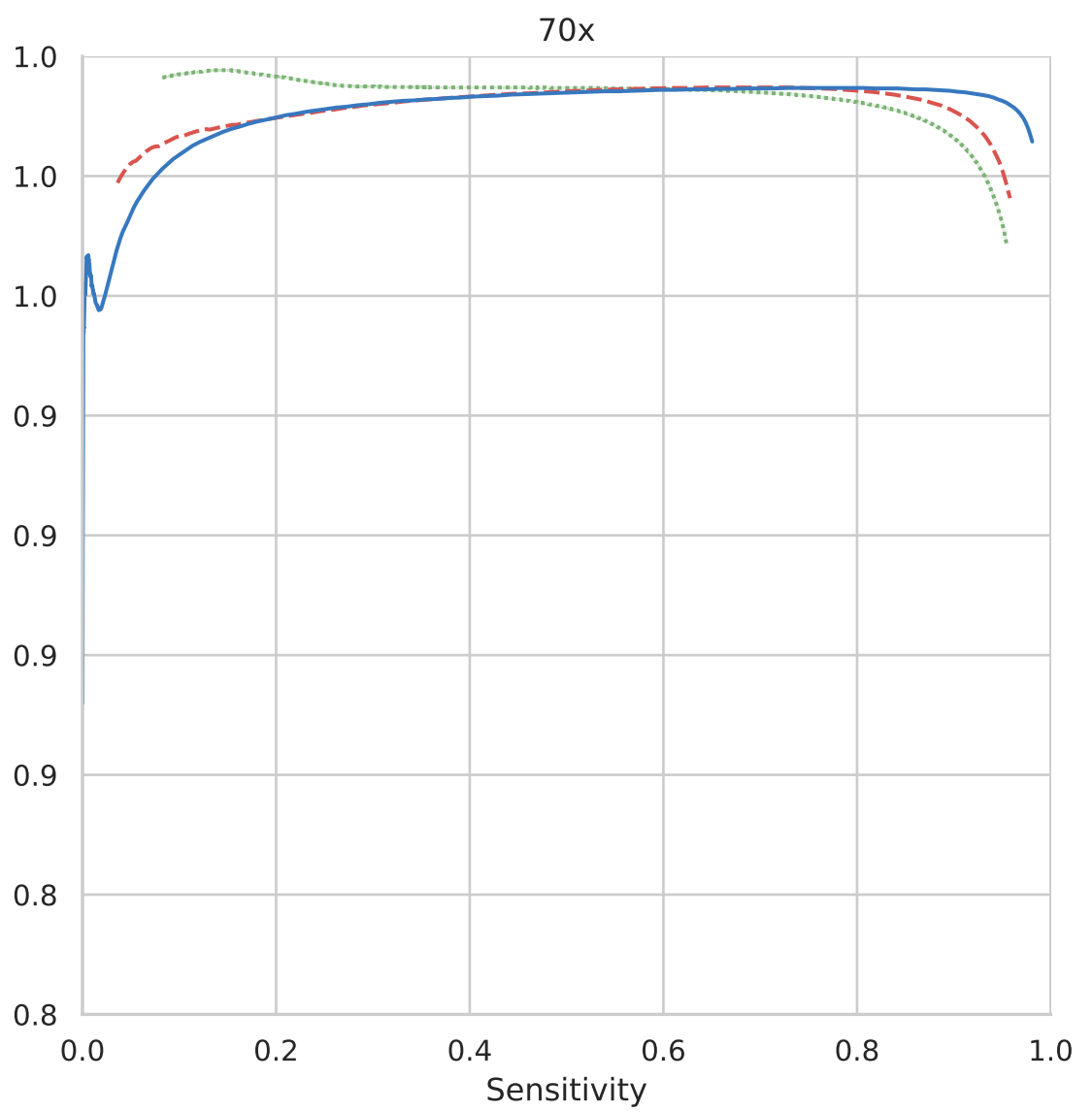

Supplement: Supplemental Material [file supp_gr.275579.121_Supplemental_Code.zip › polyploid-1.0.0/paper/supplementary/figures/synthetic-tetraploid-pr-curves.pdf]

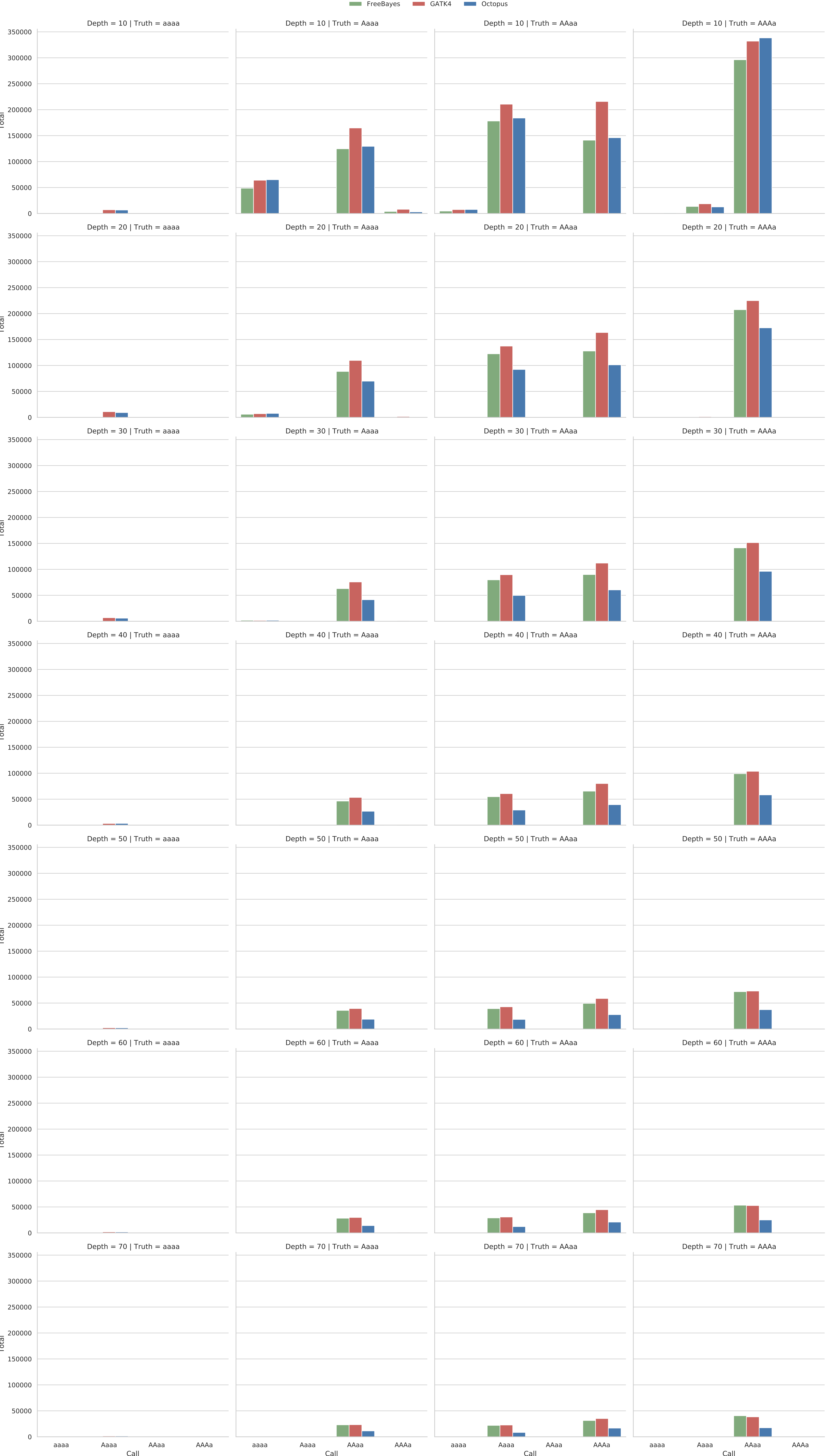

Supplement: Supplemental Material [file supp_gr.275579.121_Supplemental_Code.zip › polyploid-1.0.0/paper/supplementary/figures/tetraploid_biallelic_copy_errors.pdf]

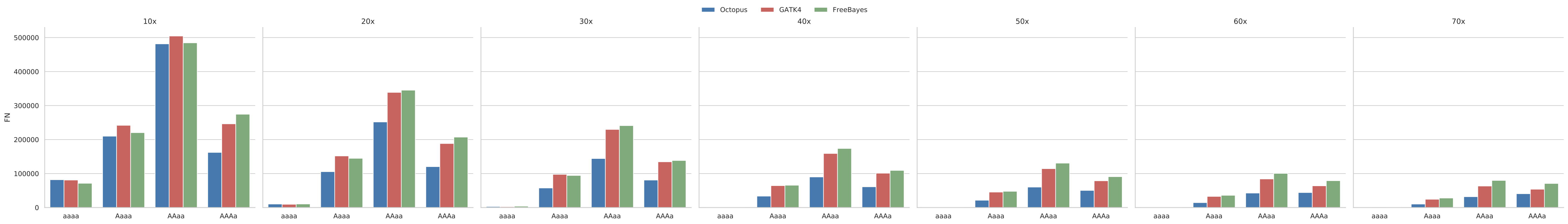

Supplement: Supplemental Material [file supp_gr.275579.121_Supplemental_Code.zip › polyploid-1.0.0/paper/supplementary/figures/tetraploid_gt_fn.pdf]

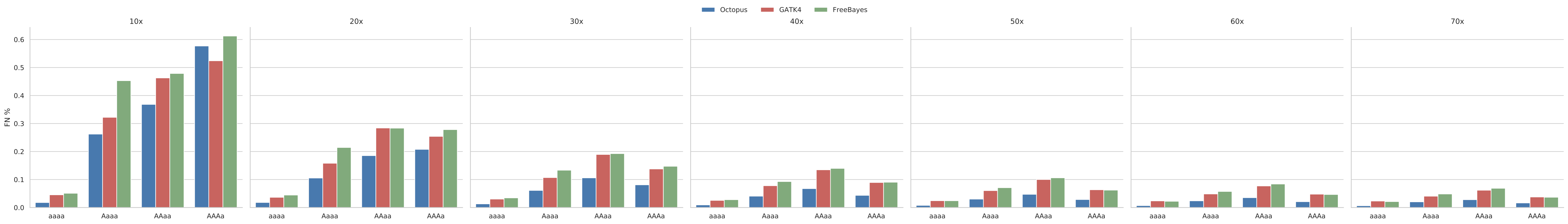

Supplement: Supplemental Material [file supp_gr.275579.121_Supplemental_Code.zip › polyploid-1.0.0/paper/supplementary/figures/tetraploid_gt_fn_perc.pdf]

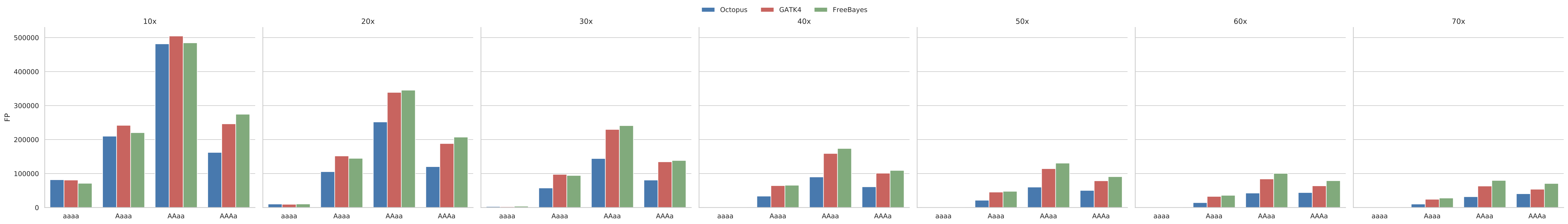

Supplement: Supplemental Material [file supp_gr.275579.121_Supplemental_Code.zip › polyploid-1.0.0/paper/supplementary/figures/tetraploid_gt_fp.pdf]
