# Supplementary material for: Benchmarking small-variant genotyping in polyploids
Source: Genome Res. 2022 Feb;32(2):403–8. doi: 10.1101/gr.275579.121 (PMC8805713; doi:10.1101/gr.275579.121)
Supplement: Supplemental Material [file supp_gr.275579.121_Supplemental_Material.pdf]

Supplemental Material

Benchmarking small-variant genotyping in polyploids

Daniel P Cooke[1,*], David C Wedge[2], and Gerton Lunter[3,1]

1. MRC Weatherall Institute of Molecular Medicine, University of Oxford, Oxford, UK.
2. Manchester Cancer Research Centre, University of Manchester, Manchester, UK.
3. Department of Epidemiology, University Medical Center Groningen, Groningen, The Netherlands.

*Correspondence should be addressed to D.P.C. (daniel.cooke@me.com)

Supplemental Figures

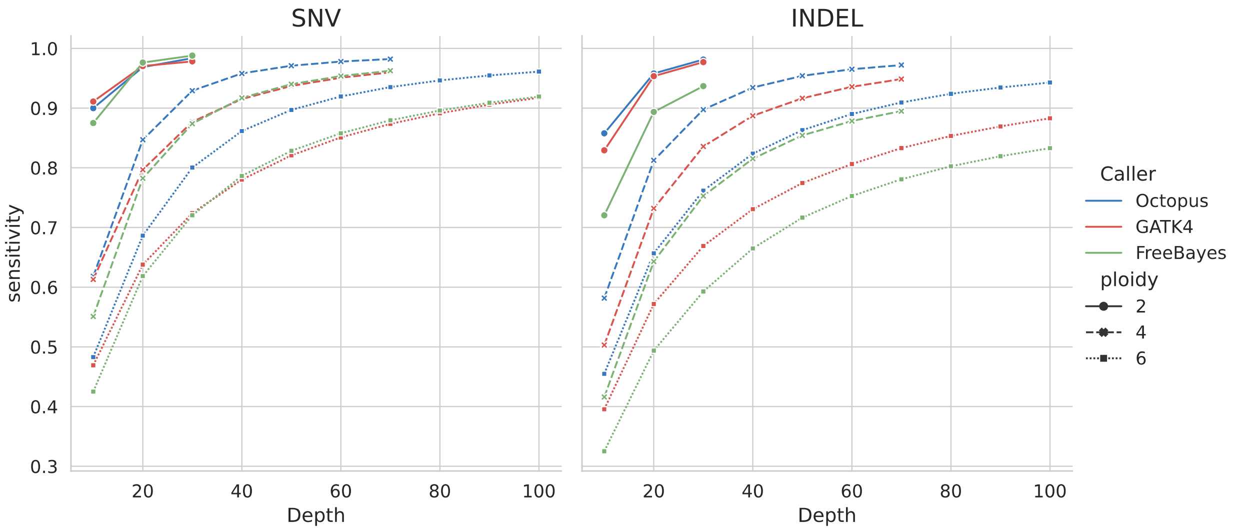

Supplemental Figure S1. Genotyping sensitivity stratified by variant type. We only present sensitivity as this can be calculated using the baseline representation only; on the other hand, precision requires counting false-positives using caller-dependent represention.

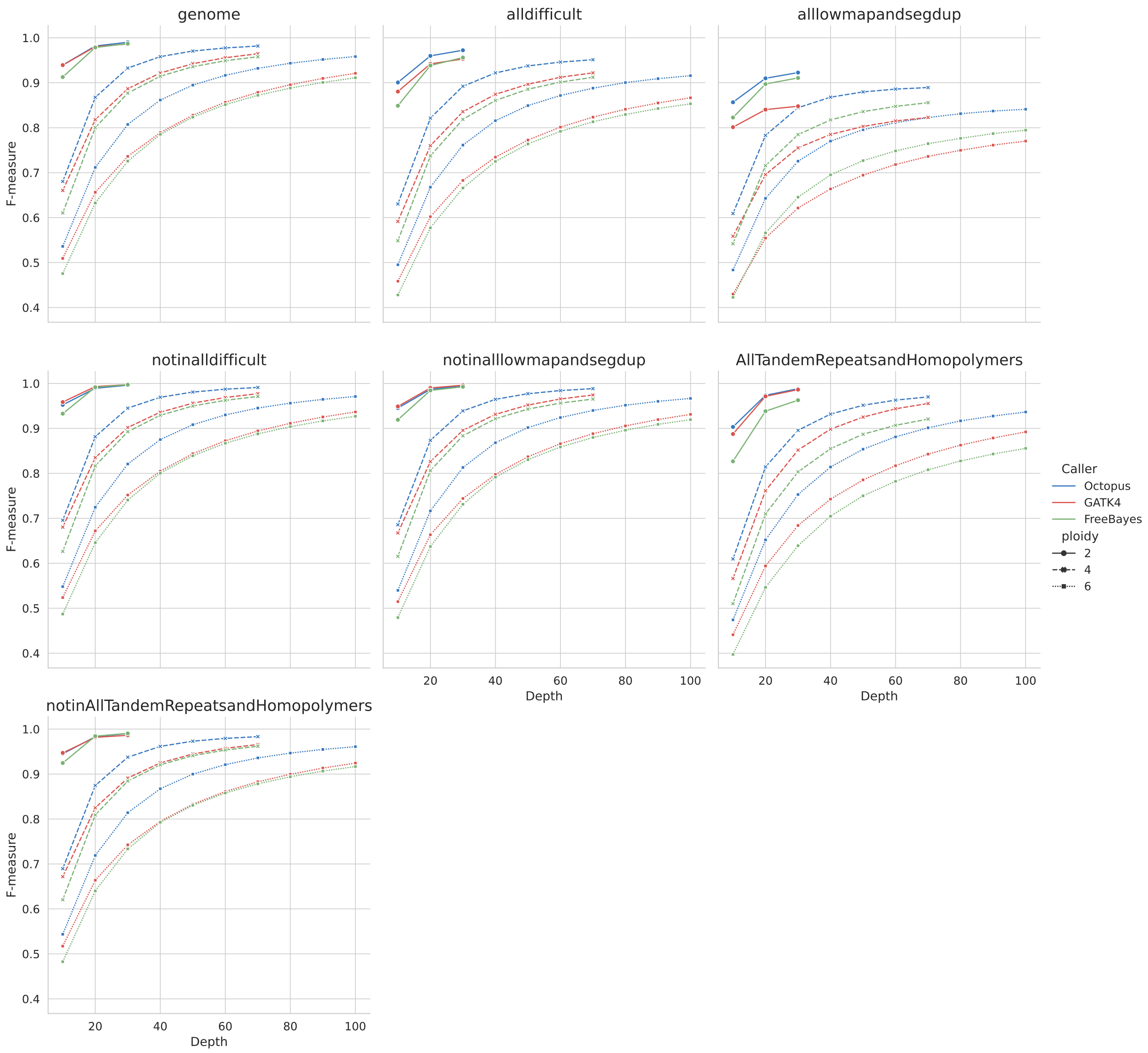

Supplemental Figure S2. Genotyping accuracy in several GIAB genome stratification’s (v2.0).

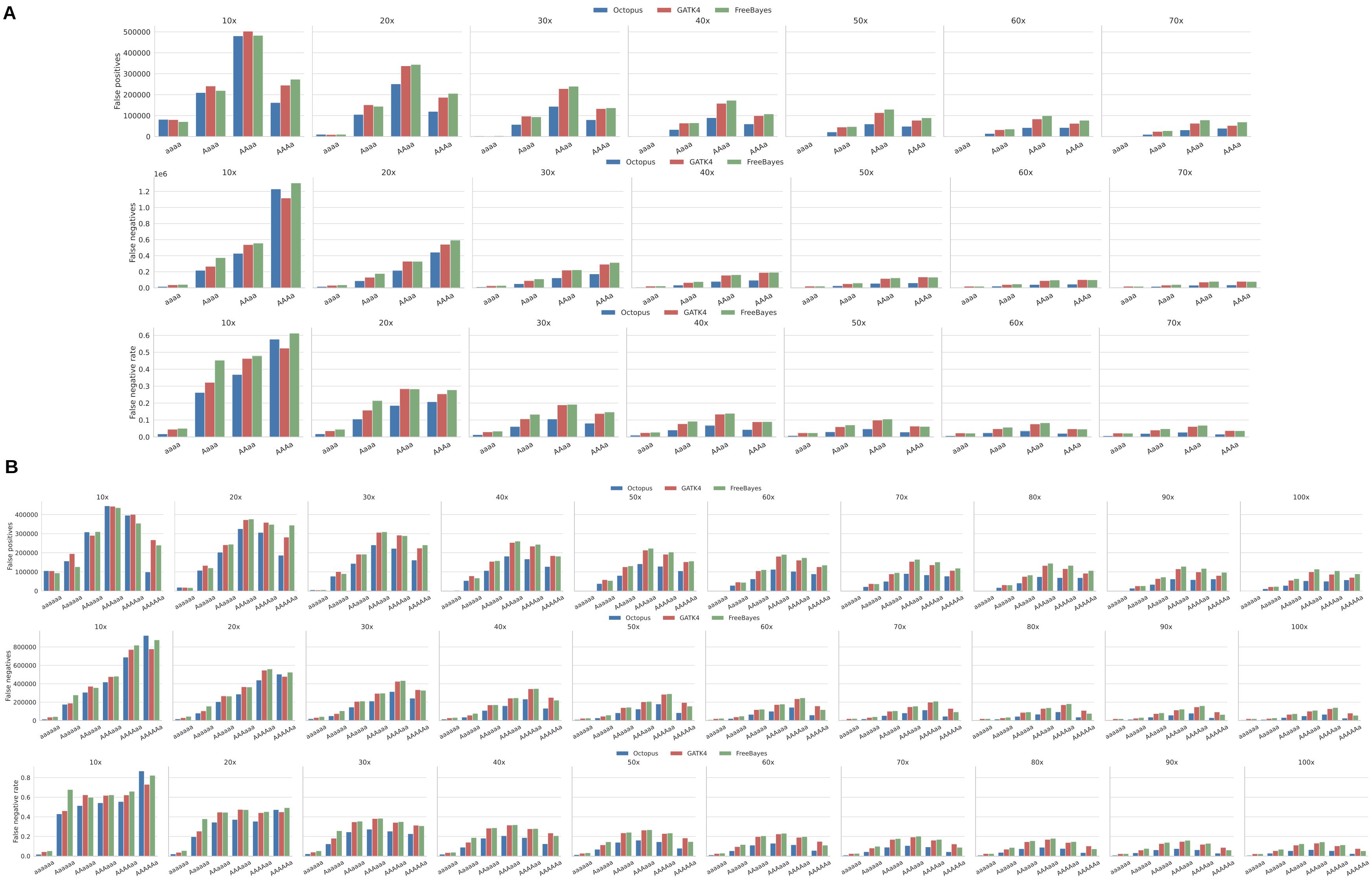

Supplemental Figure S3. Biallelic genotyping errors in synthetic polyploid samples. (A) Tetraploid. (B) Hexaploid.

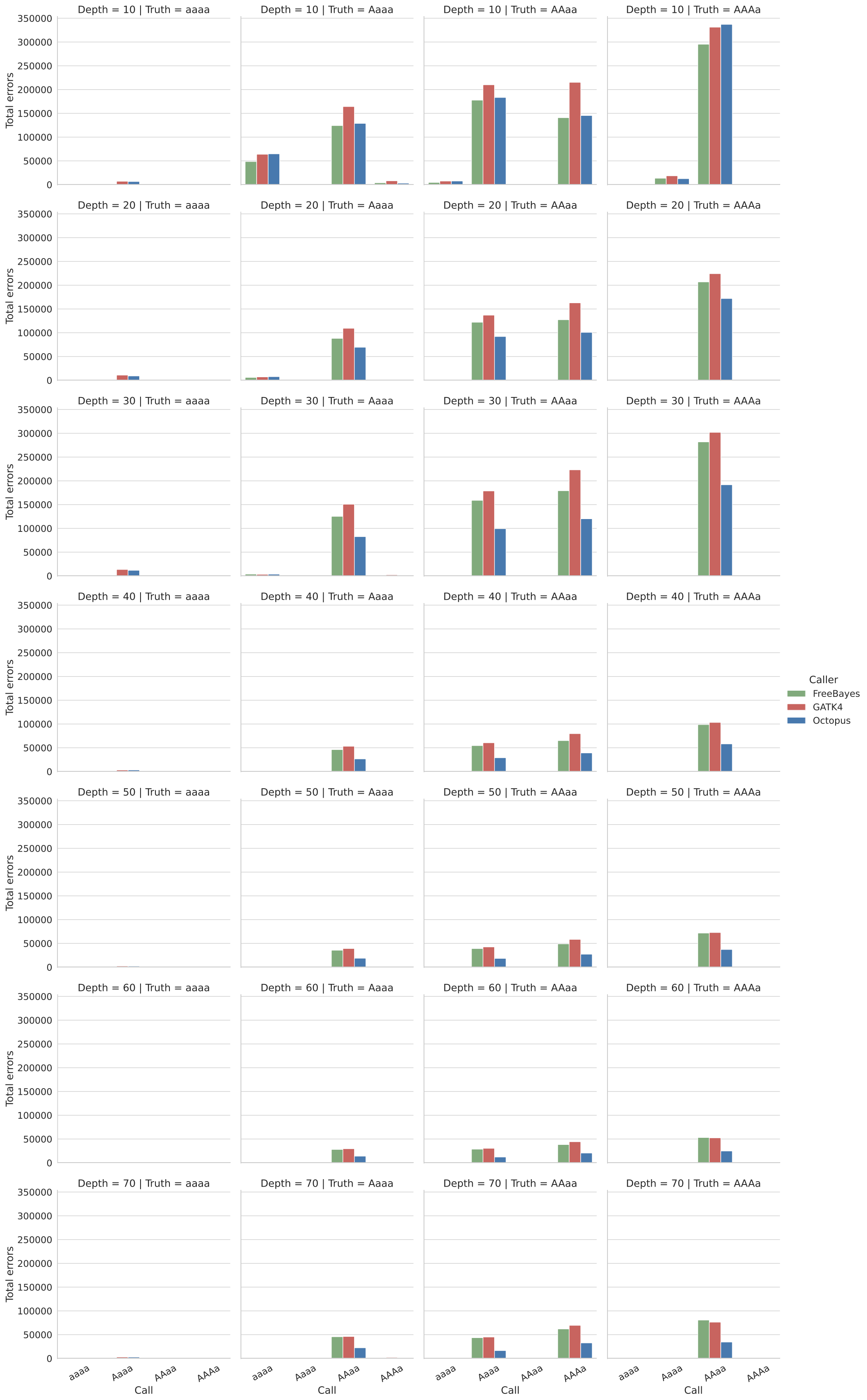

Supplemental Figure S4. Tetraploid genotyping errors at biallelic sites due to incorrect allele-specific copy number. The called (false) genotype is given on the x-axis.

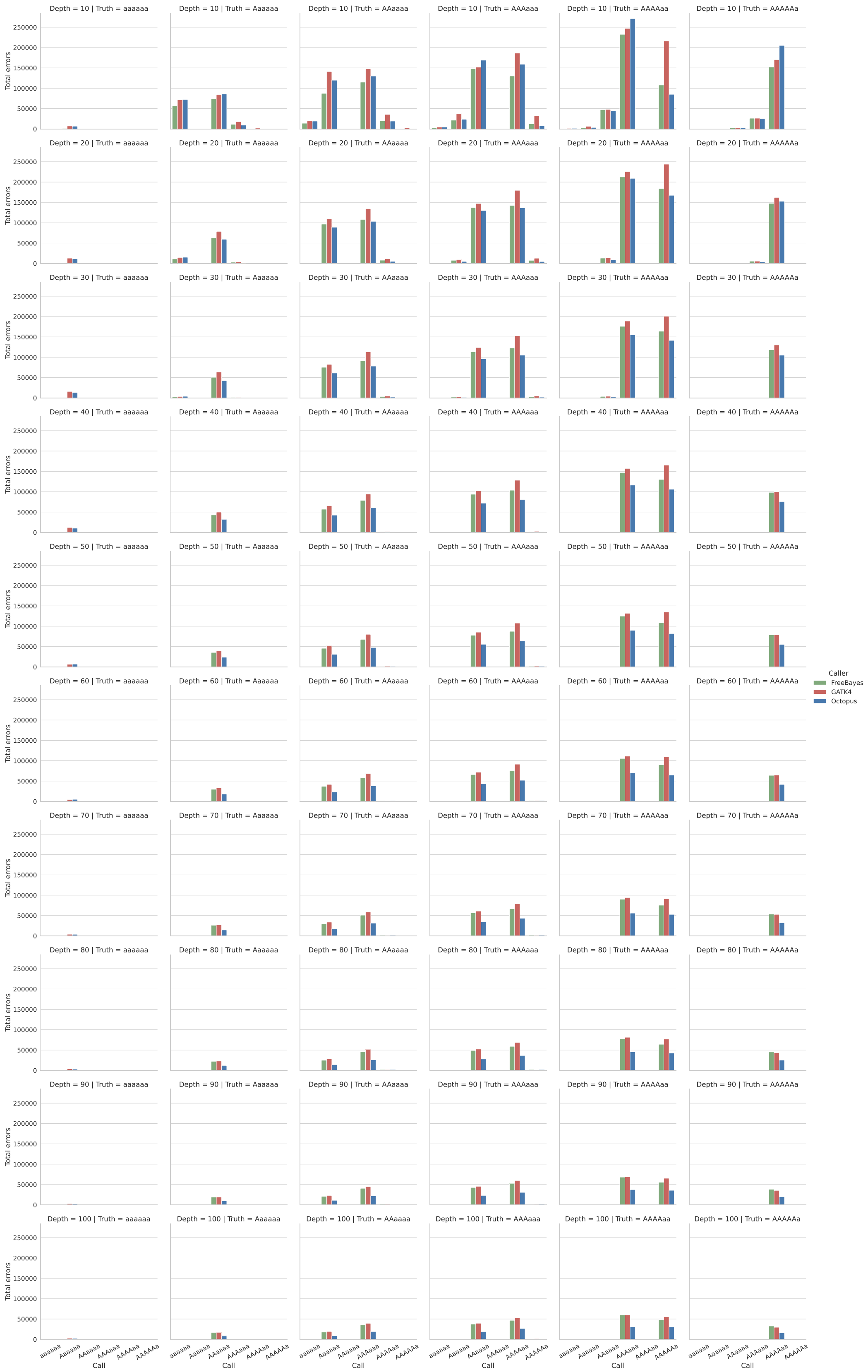

Supplemental Figure S5. Hexaploid genotyping errors at biallelic sites due to incorrect allele-specific copy number. The called (false) genotype is given on the x-axis.

A

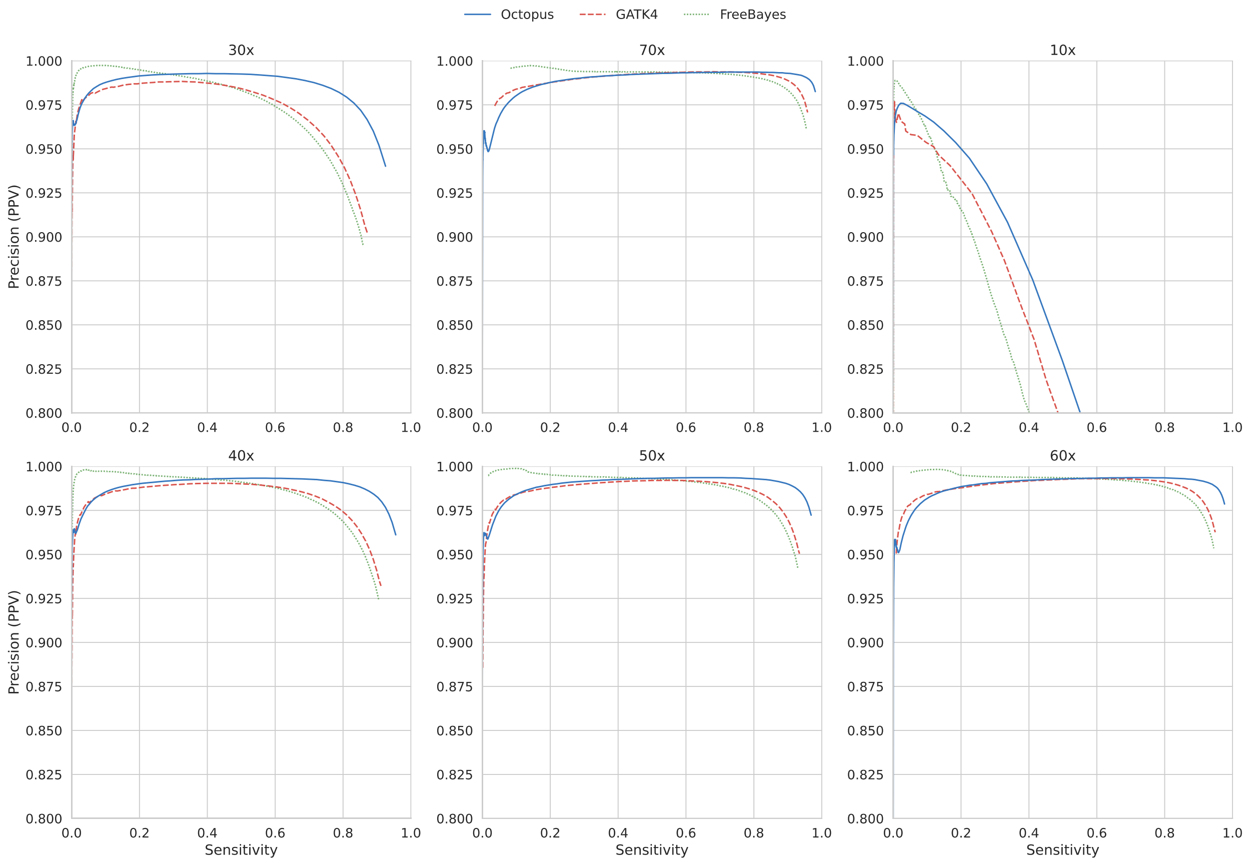

B

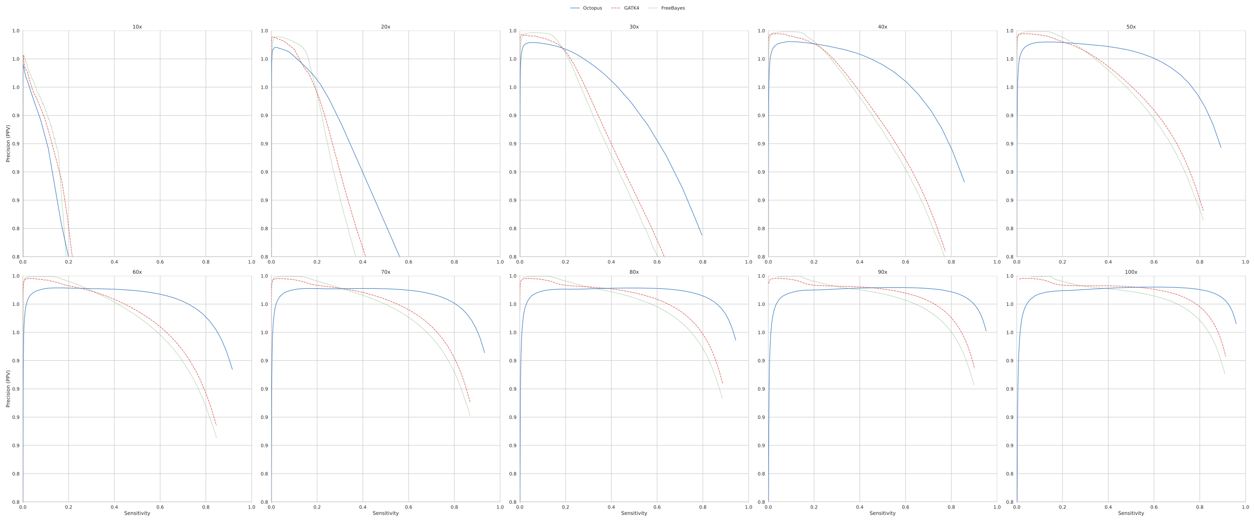

Supplemental Figure S6. Genotyping precision-recall curves in synthetic polyploid samples. GQ was used to generate curves for all samples. (A) Tetraploid. (B) Hexaploid.

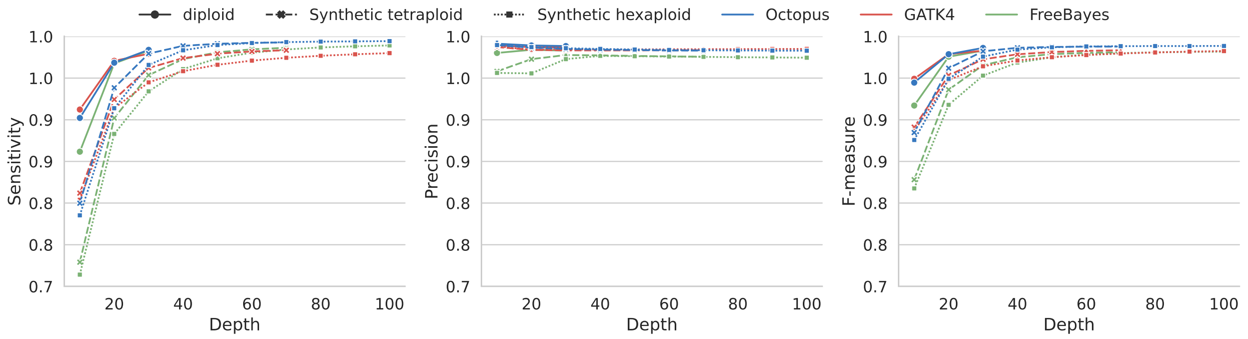

Supplemental Figure S7. Allele calling accuracy on real diploid and synthetic polyploid dataset.

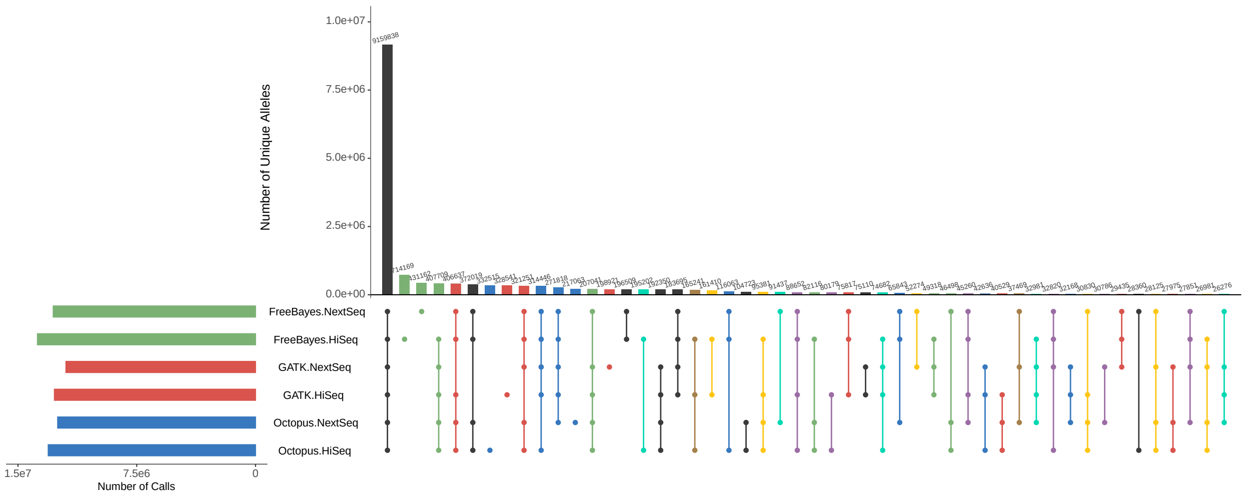

Supplemental Figure S8. Comparison of alleles called in two Illumina datasets (HiSeq and NextSeq) of banana specimen by Octopus, GATK4, and FreeBayes. UpSet plot shows callset intersections for each caller-dataset pair. The largest 50/63 intersection sets are shown. Intersections are color coded by caller discordance between the two datasets: No discordances (black), Octopus (blue), GATK4 (red), FreeBayes (green), Octopus & GATK4 (purple), Octopus & FreeBayes (cyan), GATK4 & FreeBayes (yellow), All (brown). The total number of unique alleles calls was 16,573,322.

Supplemental Tables (see excel files)

Supplemental Table S1: Performance metrics on diploid and synthetic polyploid datasets.

Supplemental Table S2: Performance metrics, stratified by SNV and INDEL, on diploid and synthetic polyploid datasets.

Supplemental Table S3: Biallelic genotyping errors in diploid and synthetic polyploid datasets.

Supplemental Table S4: Biallelic allele-specific copy number errors in diploid and synthetic polyploid datasets.

Supplemental Code

Snakemake workflow used for analysis. Copy of https://github.com/luntergroup/polyploid/archive/refs/tags/v1.0.0.zip
